# Isoreticular Contraction of Metal–Organic Frameworks Induced by Cleavage of Covalent Bonds

**DOI:** 10.1021/jacs.3c05469

**Published:** 2023-07-26

**Authors:** Yunhui Yang, Pilar Fernández-Seriñán, Inhar Imaz, Felipe Gándara, Marcel Handke, Borja Ortín-Rubio, Judith Juanhuix, Daniel Maspoch

**Affiliations:** † CSIC, and Barcelona Institute of Science and Technology, Catalan Institute of Nanoscience and Nanotechnology (ICN2), Campus UAB, Bellaterra, Barcelona 08193, Spain; ‡ Departament de Química, Facultat de Ciències, Universitat Autònoma de Barcelona, Bellaterra 08193, Spain; § Consejo Superior de Investigaciones Científicas (CSIC), Materials Science Institute of Madrid (ICMM), Calle Sor Juana Inés de la Cruz, 3, Madrid 28049, Spain; ∥ ALBA Synchrotron, Carrer de la Llum, 2, 26, Cerdanyola del Vallès, Barcelona 08290, Spain; ⊥ ICREA, Pg. Lluís Companys 23, Barcelona 08010, Spain

## Abstract

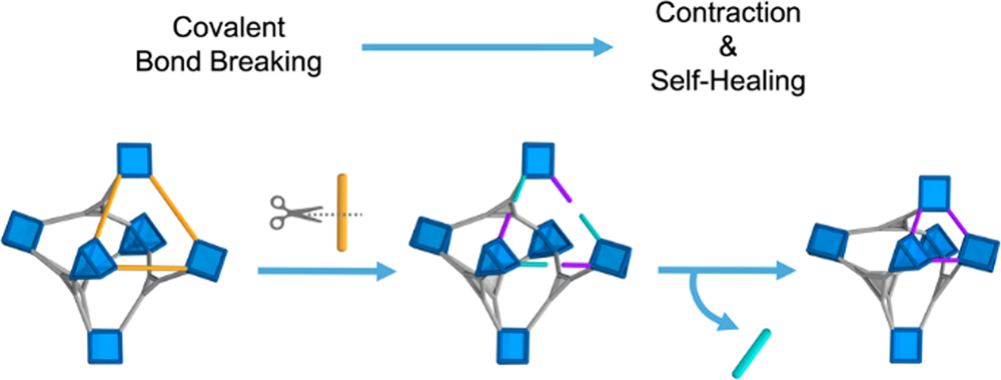

Isoreticular chemistry, in which the organic or inorganic moieties of reticular materials can be replaced without destroying their underlying nets, is a key concept for synthesizing new porous molecular materials and for tuning or functionalization of their pores. Here, we report that the rational cleavage of covalent bonds in a metal–organic framework (MOF) can trigger their isoreticular contraction, without the need for any additional organic linkers. We began by synthesizing two novel MOFs based on the MIL-142 family, (In)BCN-20B and (Sc)BCN-20C, which include cleavable as well as noncleavable organic linkers. Next, we selectively and quantitatively broke their cleavable linkers, demonstrating that various dynamic chemical and structural processes occur within these structures to drive the formation of isoreticular contracted MOFs. Thus, the contraction involves breaking of a covalent bond, subsequent breaking of a coordination bond, and finally, formation of a new coordination bond supported by structural behavior. Remarkably, given that the single-crystal character of the parent MOF is retained throughout the entire transformation, we were able to monitor the contraction by single-crystal X-ray diffraction.

## Introduction

Metal–organic frameworks (MOFs), a class of crystalline materials assembled by combining inorganic metal-based nodes with organic linkers, exhibit long-range ordered structures with permanent porosity.^1,2^ Given the nearly infinite possible permutations of their building blocks, MOFs are highly versatile: they can be designed to have distinctive features such as an exceptionally large surface area and adjustable pore sizes.

The design and synthesis of MOFs have been expanded by reticular chemistry, which provides high levels of chemical control. This is achieved mainly by either of two strategies: (i) predesign of a novel target net through judicious design of the molecular building blocks or (ii) use of a known net as a blueprint for a novel material, designed by applying the isoreticular principle.^3−6^ The primary aim of isoreticular chemistry is to tune or functionalize the organic or inorganic moieties without altering their underlying nets. Thus, it has proven invaluable for modulating the properties of MOFs and for optimizing their performance in myriad applications. Isoreticular MOFs can be synthesized by common direct synthesis^7−9^ or by postsynthetic modification (PSM). PSM-based methodologies to synthesize isoreticular MOFs include linker functionalization, transmetalation, sequential linker installation, and solvent-assisted linker exchange (SALE).^10−12^ Among these, SALE is based on substitution of a linker that bridges two metal ions/clusters, with an external linker, in the presence of a solvent. This enables generation of new isoreticular MOFs: for example, ones in which interpenetration can be controlled^13,14^ or that can exhibit nondefault topologies.^15^ In SALE, replacement is performed chiefly by using external linkers whose length is equal to or greater than that of the original linkers, thereby affording a lattice of the same or greater size.^16−18^ Alternatively, a few studies have shown that the framework can also be contracted, by using shorter linkers, although this approach has not been widely explored.^14,19,20^ The contraction of lattices can also confer the resultant MOFs^21^ with unusual properties such as negative and stepwise gas-adsorption, modulation of the radical spin stated in solid state, or stimuli responsiveness and selectivity, which can be utile for applications such as gas storage and separation, catalysis, sensing, and controlled release.^22−28^

**Figure 1:**
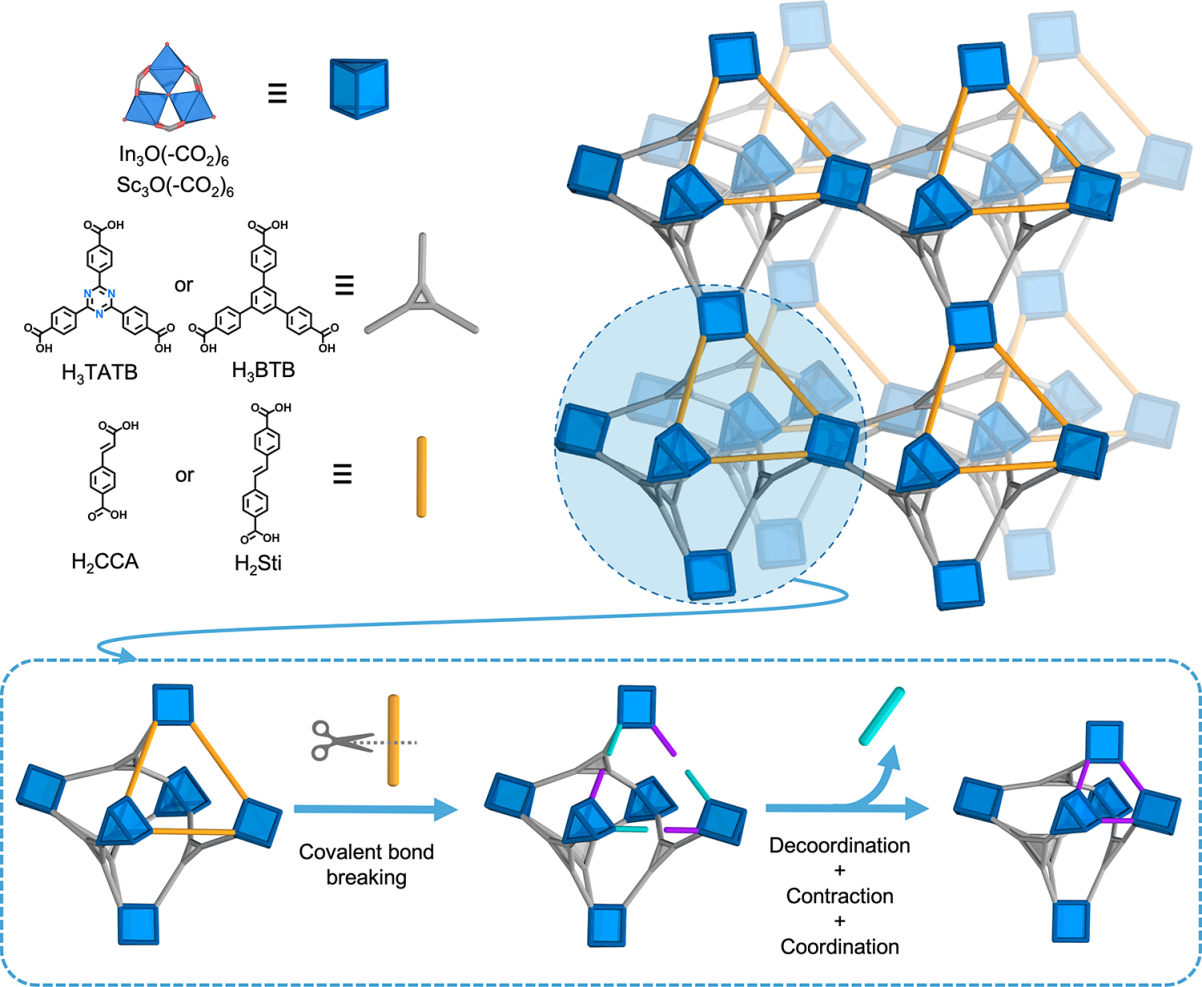
Schematic of the isoreticular contraction of (In)BCN-20B and (Sc)BCN-20C induced by cleavage of covalent bonds.

Herein, we report a new approach to isoreticular contraction of MOFs that is based on breaking of covalent bonds and does not require addition of any external linkers (Figure 1). Recently, we described clip-off chemistry, a new synthetic strategy to make new molecules and materials based on the selective, quantitative, and controlled cleavage of bonds in reticular materials.^29^ Here, we show that this concept can be applied to control the stepwise synthesis of isoreticular MOFs exhibiting contracted structures relative to their parent MOFs. To this end, we have designed two new mixed-linker parent MOFs that are isoreticular to the **nht**-(Fe)MIL-142 family:^30,31^ (In)BCN-20B and (Sc)BCN-20C (BCN stands for Barcelona Material). This new isoreticular synthetic approach begins with the cleavage of an olefinic bond of one of the bridging linkers, which splits the linker into two monocoordinated ligands. This periodic and quantitative fracture in the MOF is followed by the decoordination of one of the monocoordinated ligands, using solvents. This ligand removal instantaneously induces both contraction of the lattice and a self-healing phenomenon, which involves the migration and coordination of the monocoordinated ligand remaining in the structure to the accessible metal ions. Interestingly, this stepwise isoreticular contraction of MOFs, which involves breaking of a covalent bond, subsequent breaking of a coordination bond, and finally, formation of a new coordination bond supported by dynamic structural behavior, can be followed by single-crystal X-ray diffraction (SCXRD), as these events occur in a single-crystal to single-crystal manner.

## Results and Discussion

**Figure 2:**
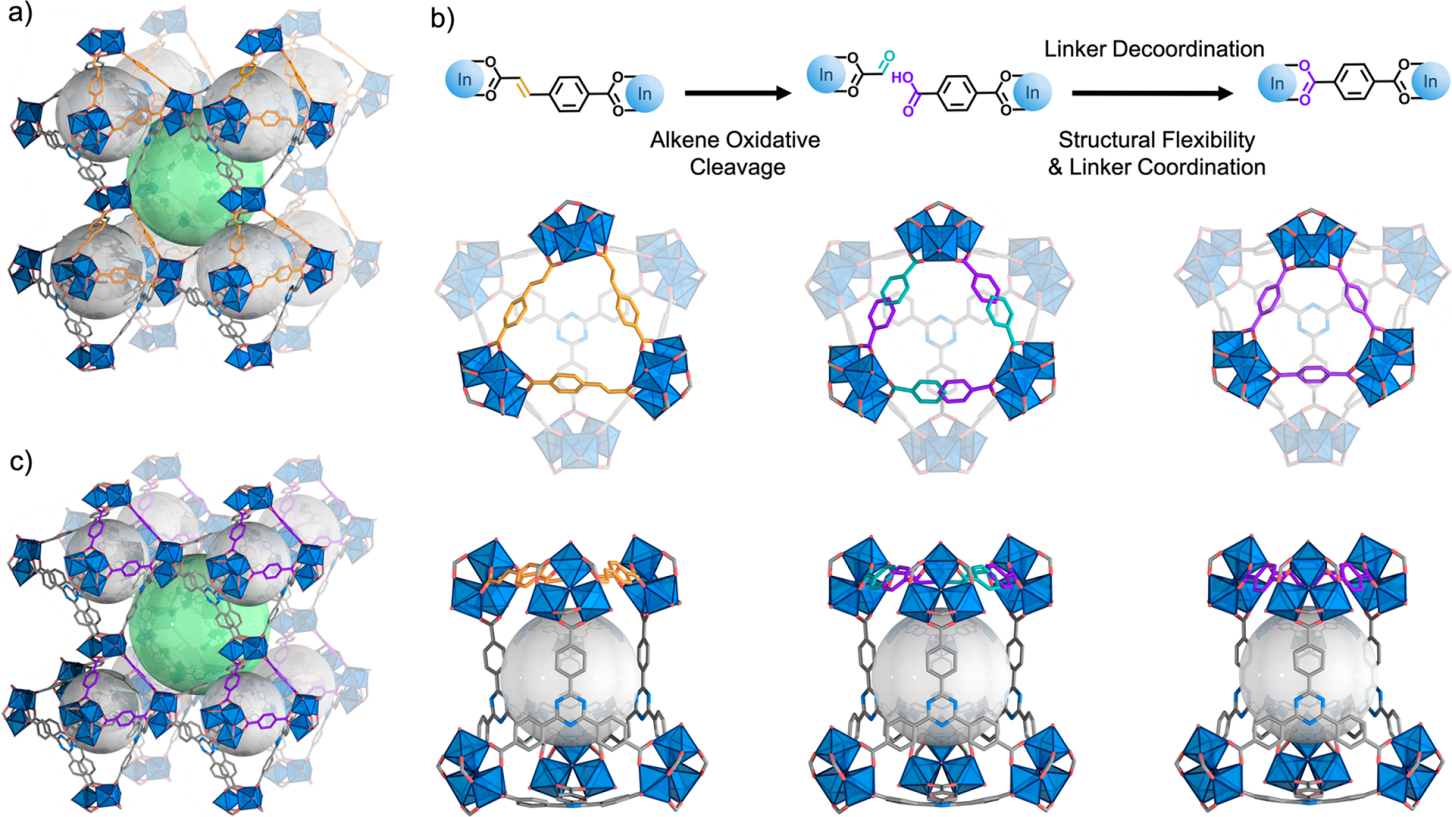
(a) Crystal structure of (In)BCN-20B. (b) Schematic of the stepwise isoreticular contraction from (In)BCN-20B (left) to (In)BCN-20B′ (middle) to (In)BCN-20A (right). Corresponding SCXRD data, revealing the octahedral cages viewed along the crystallographic *c* and *b* axes, highlighting transformation of the CCA linker (orange) to the shorter BDC linker (violet), and contraction of the triangular face. (c) Crystal structure of (In)BCN-20A.

**Figure 3:**
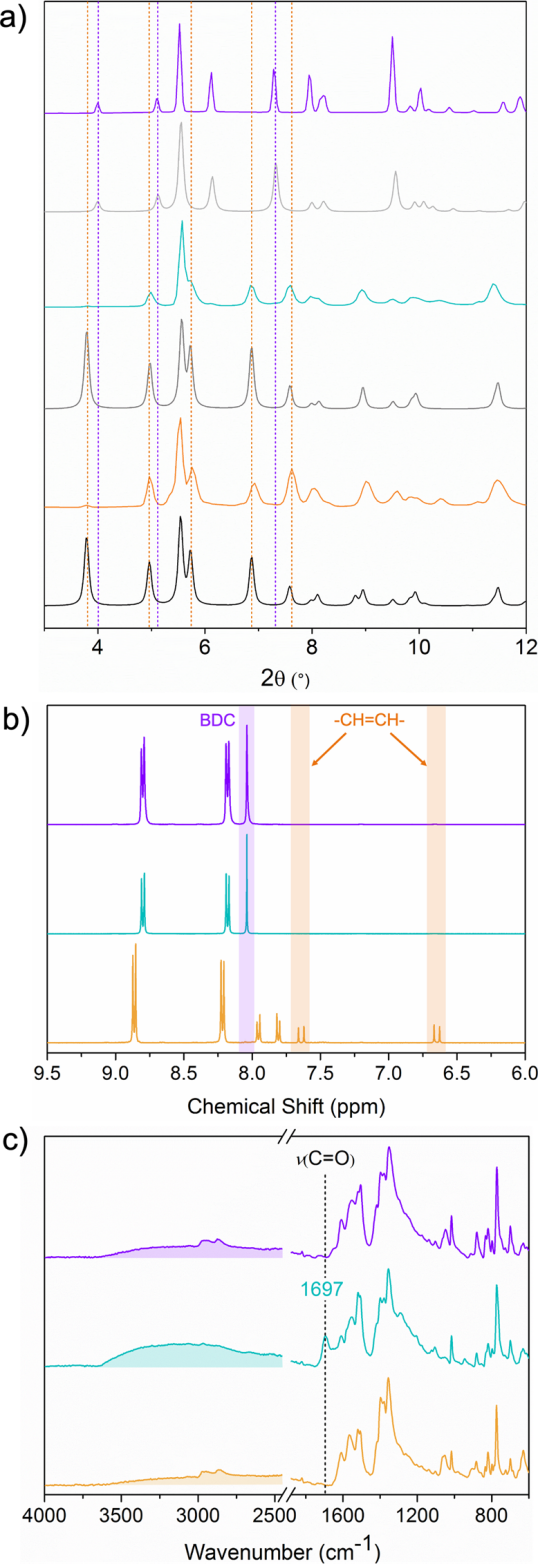
(a) PXRD patterns of simulated (black) and experimental (orange) (In)BCN-20B; simulated (gray) and experimental (cyan) (In)BCN-20B′; and simulated (light gray) and experimental (violet) (In)BCN-20A. (b) ^1^H-NMR spectra of (In)BCN-20B (orange), (In)BCN-20B′ (cyan), and (In)BCN-20A (violet). The protons from olefinic bonds and terephthalate have been highlighted in orange and violet, respectively. (c) Infrared spectra of (In)BCN-20B (orange), (In)BCN-20B′ (cyan), and (In)BCN-20A (violet).

We began by synthesizing (In)BCN-20B. First, a dispersion of In(NO_3_)_3_·*x*H_2_O, 4,4′,4″-(1,3,5-triazine-2,4,6-triyl)tribenzoic acid (H_3_TATB) and 4-carboxycinnamic acid (H_2_CCA) in *N*,*N*-dimethylformamide (DMF) and HNO_3_ (3.5 M in DMF) was heated at 120 °C for 30 h. Next, the resulting hexagonal prismatic crystals were analyzed by SCXRD, which confirmed formation of a 3D framework isoreticular to **nht**-(Fe)MIL-142B. (In)BCN-20B crystallizes in a trigonal lattice with an *R*3̅*c* (No. 167) space group, with unit cell parameters of *a* = *b* = 30.820 and *c* = 95.540 Å (Table S1). The 3D structure of (In)BCN-20B is formed by vertex-sharing distorted octahedral cages, with an underlying **nht** topology (Figure 2a). Each cage is formed by six trigonal prismatic In_3_O(−CO_2_)_6_ clusters located at vertices, which are connected by four tritopic TATB linkers and three ditopic CCA linkers. Note that these CCA linkers are symmetrically disordered about an inversion center, which results in similarity of spatial occupation between the dislocated aromatic rings of the CCA linker and those of a naphthalene molecule (Figures S1 and S2). The location of the two types of linkers within the cage defines one triangular face, whose edges are occupied by three CCA linkers (Figure 2b, left and Figure S3), and four triangular faces, each of which is occupied by one TATB linker. This means that each of the three remaining faces shares one of the edges occupied by a CCA linker with the first triangular face, whereas the other two edges are unoccupied. The overall framework of (In)BCN-20B shows a 2-fold interpenetrated structure, in which two catenane-like octahedral cages from two different **nht** nets interlocked via one of their pure TATB triangular faces (Figure S4). Further characterization of (In)BCN-20B confirmed its phase purity, as the experimental powder-XRD (PXRD) pattern matched the simulated one (Figure 3a). Furthermore, the ^1^H-NMR spectrum of the digested sample contained the expected TATB/CCA ratio of 4:3 (Figure S6).

Having synthesized (In)BCN-20B, we then cleaved all its alkene bonds and subsequently converted them into aldehyde/carboxylic acid groups via a solid–gas ozonolysis reaction.^29,32,33^ First, 20 mg of single crystals of solvent-free (In)BCN-20B were packed into a plastic tube. Next, dry ozone was flowed (∼15 g/Nm^3^) continuously through the sample for 35 min. Finally, the resultant crystals were analyzed by SCXRD, showing that ozonized (In)BCN-20B′ crystallized in a trigonal lattice with an *R*3̅*m* (No. 166) space group, having unit cell parameters of *a* = *b* = 30.830 and *c* = 47.592 Å (Table S2). Overall, (In)BCN-20B′ exhibited a related 3D structure to that of (In)BCN-20B but with evident changes in its connectivity through the CCA linkers. Indeed, SCXRD data confirmed the integrity of the inorganic In-oxo clusters and of the TATB linkers. However, at the positions initially occupied by the CCA linkers, the similarity in spatial occupation between the dislocated aromatic rings of the CCA linker and the naphthalene molecule was lost (Figure 2b, middle). This observation clearly suggested that the CCA linkers no longer existed.

To confirm the oxidative cleavage of CCA linkers into the expected monocoordinated glyoxylate and single deprotonated terephthalic acid (BDC) ligands (Figure 2b), we analyzed a digested (In)BCN-20B′ sample by ^1^H NMR and then compared the resulting spectrum to that of the starting (In)BCN-20B (Figures 3b and S6, S8). The spectrum of the digested (In)BCN-20B showed the characteristic peaks of the olefinic protons of CCA at δ = 7.64 and δ = 6.65 ppm. In contrast, the spectrum of the digested (In)BCN-20B′ corroborated the disappearance of this olefinic signal. It also revealed the disappearance of the phenyl (δ = 7.94 and δ = 7.81 ppm) and the carboxylic acid (δ = 13.06 ppm) protons of the CCA but did show the characteristic signals for the terephthalic acid (BDC, δ = 8.04 ppm). Unfortunately, the aldehyde proton of the glyoxylic acid was not detected, presumably due to the fact that its boiling point (111 °C) is lower than the temperature (120 °C) at which the digestion took place overnight. However, the presence of glyoxylic acid was confirmed by electrospray ionization mass spectrometry (ESI-MS) analysis in a negative ion mode using a (In)BCN-20B′ sample digested under milder conditions (HF in DMSO at room temperature). The ESI-MS spectrum showed a peak at *m*/*z* = 72.99, which matches the molecular mass of the deprotonated glyoxylate [C_*2*_HO_3_]^*–*^ (*m*/*z* = 73.03; Figure S9). These results clearly indicated that the olefinic bond of the ditopic CCA linkers had been quantitatively cleaved by ozonolysis and subsequently converted into two monocoordinated ligands: a single deprotonated BDC and a glyoxylate. Supporting this oxidative cleavage reaction, the FTIR spectrum of the ozonated (In)BCN-20B′ exhibited a more intense C=O stretch band at 1697 cm^–1^ relative to that in the spectrum of the non-ozonated (In)BCN-20B. Moreover, it showed enhanced, broad absorbance at around 3300 cm^–1^, indicative of large perturbations caused by H-bonded hydroxyl groups of the free carboxylic acid groups (Figures 3c and S10).^34,35^ Next, we performed PXRD on a bulk sample of ozonated (In)BCN-20B′ to confirm its crystallinity and phase purity. The resulting PXRD pattern matched the one calculated from the crystal structure (Figures 3a and S11), confirming that the cleavage of alkene bonds via ozonolysis did not compromise the crystallinity throughout the bulk sample. Together these results confirmed the quantitative cleavage of CCA linkers and the consequent formation of a new structure with less connectivity among its trimeric In^3+^ clusters. Indeed, analysis of the topology of (In)BCN-20B′ using the ToposPro 5.3.3.5 software,^36^ and considering TATB linkers as 3-connected points of extension (3-c), and In_3_O(−CO_2_)_6_ clusters as nodes that have four coordination points (4-c), revealed formation of a 2-fold interpenetrated MOF with two independent underlying trinodal (3,3,4)-c nets (topological code **3,3,4T22**, Figure S12).

Having demonstrated that ditopic CCA linkers could be cleaved into two monocoordinated ligands, we then explored the possibility of substituting these monocoordinated ligands with solvent molecules. First, crystals of (In)BCN-20B′ were incubated in DMF for 1 week at room temperature. Next, the resultant crystals were analyzed by SCXRD, which revealed that a new 3D structure (In)BCN-20A had been formed (Figure 2c). This new MOF crystallizes in the trigonal lattice with the space group *R*3̅*m* (No. 166) and lattice parameters of *a* = *b* = 28.783 and *c* = 47.708 Å (Table S3). (In)BCN-20A, which is isostructural to **nht**-(Fe)MIL-142A,^30^ recovers the initial 3D **nht** framework formed by vertex-sharing distorted octahedral cages. However, its cages are instead formed by six In_3_O(−CO_2_)_6_ clusters connected by four tritopic TATB linkers and three ditopic BDC linkers, the latter occupying the analogous position of the CCA linkers in (In)BCN-20B (Figure 2b, right).

The phase transition from (In)BCN-20B′ to (In)BCN-20A unambiguously unveiled a self-healing behavior, which we envisaged occurs in three steps within the pores. First, DMF molecules enter the pores and replace the glyoxylate linkers. Here, the release of glyoxylate from the MOF was experimentally confirmed by analyzing the DMF supernatant resulting from the incubation process by ESI-MS, from which the characteristic peak at *m*/*z* = 72.99 was detected (Figure S13). Second, the decoordination of glyoxylate causes the framework to act dynamically. In the third and final step, this behavior leads to coordination of the free carboxylic acid group of each BDC linker to the In^3+^ metal sites that were previously occupied by the glyoxylate. Accordingly, the BDC linker would have to migrate a distance of ∼2.4 Å.

To further evaluate the degree of the aforementioned flexibility, we compared the unit cell volumes of the three MOFs: the initial (In)BCN-20B, the ozonated (In)BCN-20B′, and the self-healed (In)BCN-20A (Table S4). From (In)BCN-20B to (In)BCN-20B′, the unit cell volume only shrunk by 0.3%. However, from (In)BCN-20B′ to (In)BCN-20A, the cell volume decreased by 12.6%, an obvious volumetric contraction. We reasoned that the movements involving this dynamic behavior could also be studied by comparing the structural changes in the octahedral cage units (Figures 2b and S14). In the octahedral cage of the initial (In)BCN-20B, the length (note: all lengths were calculated starting from the carbon atoms of the carboxylate groups of the linkers) of the edges of the face constructed with CCA linkers is 8.0 Å, and the average length of the remaining edges is 12.1 Å. As we had expected, cleavage of the CCA linkers barely affected this octahedral cage unit: the length of the edges of the face occupied by the cleaved CCA linkers only increased to 8.2 Å. We attributed this slight increase to the steric hindrance effects of both the BDC and the glyoxylate linkers. In (In)BCN-20A, this length decreases significantly, down to 5.7 Å, because the BDC linker is much smaller than both the initial and the cleaved CCA linkers. Contrariwise, this contraction barely affects the other edges involving the TATB linker, exhibiting an average length of 12.0 Å.

Given that the structure of (In)BCN-20A differs markedly from that of (In)BCN-20B or (In)BCN-20B′, we studied whether this transformation would occur throughout the bulk sample, using PXRD. Supporting a homogeneous transformation, the PXRD pattern of (In)BCN-20A matched the one calculated from the corresponding structure determined by SCXRD (Figures 3a and S15). Comparing the PXRD patterns of (In)BCN-20A and (In)BCN-20B, we found that the diffraction peak of (In)BCN-20B at 2θ = 3.8° which corresponds to the (102̅) crystallographic plane being shifted to a higher angle (2θ = 5.1°) in the same plane of (In)BCN-20A, thus confirming the compression of the framework. Phase homogeneity was also studied by FTIR and ^1^H NMR (Figures S16 and S17). The FTIR spectrum revealed that the stretching band of characteristic carbonyl groups (1697 cm^–1^) and the broad absorbance at around 3300 cm^–1^ had been dramatically attenuated, identically to that of the initial (In)BCN-20B (Figure 3c). The ^1^H NMR spectrum confirmed the expected TATB/BDC ratio of 4:3.

We would like to highlight that all our attempts to transform (In)BCN-20B into (In)BCN-20A by direct linker exchange of CCA by BDC were unsuccessful (see Section 2 of the Supporting Information). This suggests that covalent bond cleavage of the CCA linker highly facilitates the internal restructuration of the framework, involving both flexible and self-healing behavior.

**Figure 4:**
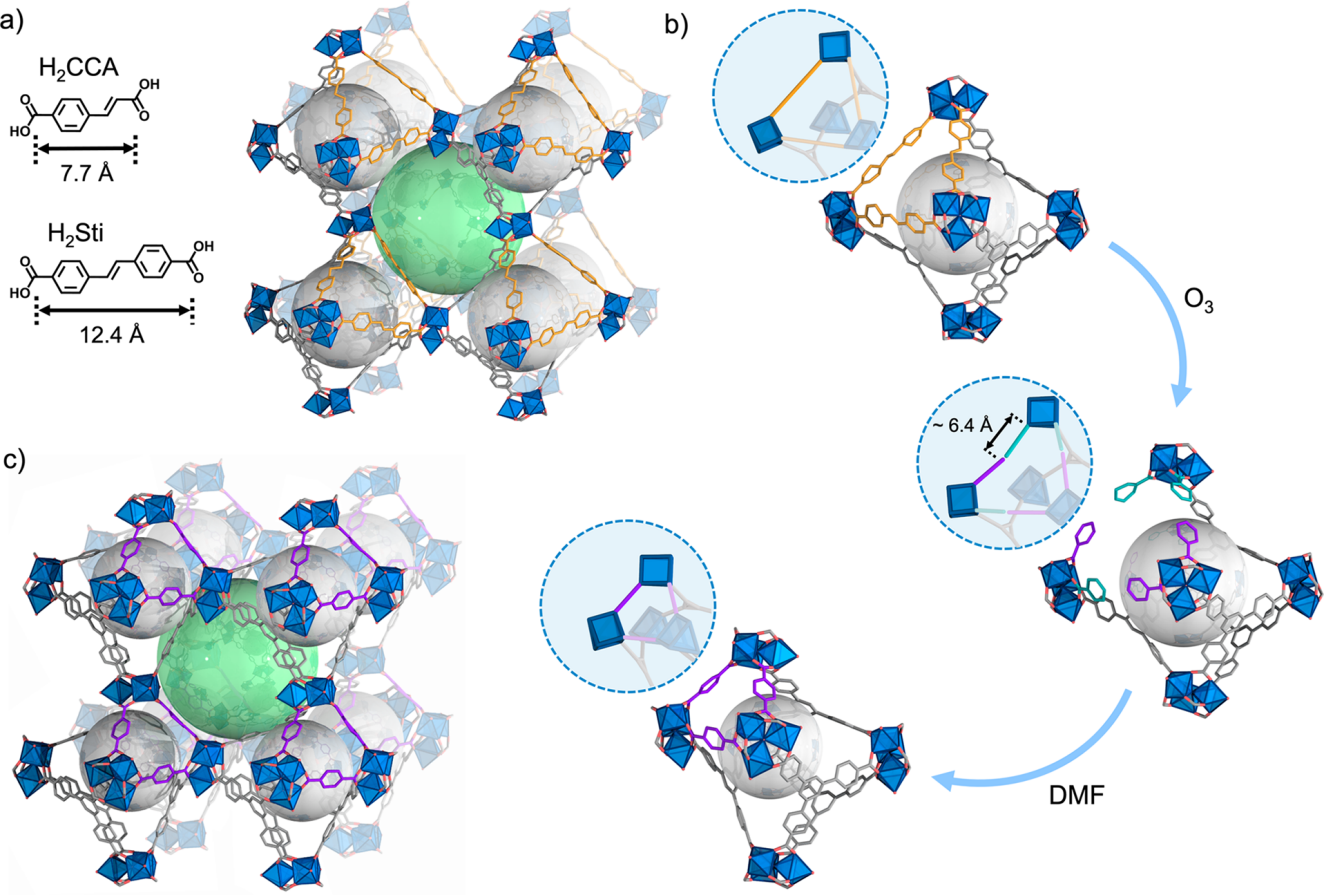
(a) Crystal structure of (Sc)BCN-20C. (b) Schematic and corresponding SCXRD structures of the stepwise isoreticular contraction from (Sc)BCN-20C (top) to (Sc)BCN-20C′ (middle) to (Sc)BCN-20A (down). (c) Crystal structure of (Sc)BCN-20A.

Seeking to investigate whether such behavior could occur in an isoreticular framework with greater distances between metal clusters, we substituted the CCA linker with the 4,4′-stilbenedicarboxylic acid (H_2_Sti) linker, which is longer (Figure 4a). However, all our attempts at synthesizing the isostructural MOF using In^3+^ were unsuccessful. Fortunately, we were able to synthesize its Sc^3+^ analogue using the (ditopic) Sti linker and the (tritopic) 1,3,5-tris(4-carboxyphenyl)benzene (BTB) linker (Figure 4a). (Sc)BCN-20C crystallizes in the monoclinic symmetry with a *C*2/*c* space group (No. 15) and lattice parameters of *a* = 60.279, *b* = 34.723, and *c* = 37.136 Å (Table S5). As its analogues, it contains a 2-fold interpenetrated structure with two underlying **nht** nets (Figure S18). Note that the two nets are interconnected by formate linkers that can be formed upon decomposition of DMF aided by HNO_3_.^31,37^ These nets are built up from vertex-sharing distorted octahedral cages, which are larger than those of (In)BCN-20B or (In)BCN-20A. Their main difference lies in the edges of the triangular face defined by three Sti linkers, whose length is increased to 12.2 Å. The other edges of the octahedra are slightly longer (12.3 Å) than those of (In)BCN-20B. The phase purity of (Sc)BCN-20C was confirmed by PXRD, whose pattern matched the one simulated from SCXRD data (Figures S19 and S20), and by ^1^H NMR, the spectrum confirmed the expected BTB/Sti ratio of 4:3 (Figure S23).

Next, we performed a cleavage experiment on the Sti linkers similar to the previous experiment on the CCA linkers (Figure 4b). Thus, ozone was flowed (∼15 g/Nm^3^) continuously through a crystalline sample of solvent-free (Sc)BCN-20C for 5 min. The quantitative cleavage of Sti linkers and conversion of the resultant fragments to the expected monocoordinated HBDC and 4-formylbenzoate (4-FBA) were corroborated by ^1^H NMR, SCXRD, PXRD, and FTIR (Table S6, Figures S21, S24, S25, and S29). Additionally, SCXRD data revealed that the structure of (Sc)BCN-20C’ is very similar to that of the initial (Sc)BCN-20C, with the corresponding octahedral cage units having similar dimensions. However, we observed one significant difference: benzoate ligands—most likely, 4-FBA linkers with highly disordered aldehyde groups—bridging the two interpenetrated nets and partially substituting the formate ligands. This observation evidenced the mobility of these monocoordinated ligands inside the pores upon cleavage of the ditopic linkers.

Finally, endeavoring to remove the 4-FBA ligands and induce the dynamic and self-healing behavior for the formation of the isoreticular (Sc)-BCN-20A, we immersed (Sc)BCN-20C′ in DMF for a week. Interestingly, SCXRD of the resulting crystals confirmed the formation of this MOF (Table S7, Figures S18, S19, S22, and S26–S29), whose structure is isostructural to that of (In)BCN-20A (Figure 4b,c). (Sc)-BCN-20A crystallizes in a trigonal system with the space group *R*3̅*c* (No. 167) and lattice parameters of *a* = *b* = 28.682 and *c* = 95.606 Å. Accordingly, through this dynamic, self-healing phenomenon, the cell volume had been dramatically compressed by 30.7% (Table S8 and Figure S31). In this process, all BDC linkers traveled a distance of ∼6.4 Å to coordinate to the Sc^3+^ metal sites, shrinking one of the triangular faces of the cages from 12.2 to 6.1 Å.

## Conclusions

In conclusion, we have shown that the cleavage of covalent bonds within bridging organic linkers in an MOF can trigger a series of chemical and structural dynamic processes that drive the formation of an isoreticular contracted MOF. Remarkably, as the single-crystal character of the parent MOF is retained throughout the entire isoreticular transformation, we were able to use SCXRD to obtain invaluable crystallographic snapshots of the stepwise processes. Initially, applying our concept of clip-off chemistry, we were able to cleave each bridging alkene-containing organic linker into two monocoordinated ligands via ozonolysis, thereby disconnecting two metal clusters. Among these monocoordinated ligands, one is terminated with a carboxylic acid group, whereas the other ends in an aldehyde group. As this latter ligand is weaker, we were able to decoordinate it from the metal center by simply treating the MOF with DMF. This removal process conferred the structure with dynamic behavior that involved the migration and subsequent coordination of the other monocoordinated ligand with a free carboxylic acid group to the open metal site. In what we have called “self-healing behavior”, the metal clusters that had been disconnected during cleavage of the linker bond reconnected to form a new MOF isoreticular to the initial one, with a contracted structure. Thus, the complete transformation involves breaking of covalent bonds, breaking and formation of coordination bonds, and contraction of the crystal structure, with reductions in the cell volume of up to 30.7%, all occurring in a single-crystal to single-crystal manner. Overall, these stepwise isoreticular transformations exemplify the rich chemistry that can be done inside MOF pores. They also underscore the potential of clip-off chemistry (i.e., breaking of covalent bonds) to discover new phenomena in MOFs and to synthesize new MOFs or other materials.

## Supporting Information Available

Detailed syntheses, optical microscopy images, PXRD diffractogram, FT-IR, ESI-MS, NMR, and crystallographic details (PDF)
