# Supplementary material for: Isoreticular Contraction of Metal–Organic Frameworks Induced by Cleavage of Covalent Bonds
Source: J Am Chem Soc. 2023 Jul 26;145(31):17398–405. doi: 10.1021/jacs.3c05469 (PMC10416301; doi:10.1021/jacs.3c05469)
Supplement: Supplementary file 1 — ja3c05469_si_001.pdf [file ja3c05469_si_001.pdf]

Supporting Information for

Isoreticular Contraction of Metal-Organic Frameworks Induced by Cleavage of Covalent Bonds

Yunhui Yang[1,2], Pilar Fernández-Seriñán[1,2], Inhar Imaz*[,1,2], Felipe Gándara[3], Marcel Handke[1,2], Borja Ortín-Rubio[1,2], Judith Juanhuix[4] and Daniel Maspoch*[,1,2,5]

[1] Catalan Institute of Nanoscience and Nanotechnology (ICN2), CSIC, and Barcelona Institute of Science and Technology, Campus UAB, 08193 Bellaterra, Barcelona, Spain.

[2] Departament de Química, Facultat de Ciències, Universitat Autònoma de Barcelona, 08193 Bellaterra, Spain.

[3] Consejo Superior de Investigaciones Científicas (CSIC), Materials Science Institute of Madrid (ICMM), Calle Sor Juana Inés de la Cruz, 3, 28049 Madrid, Spain.

[4] ALBA Synchrotron, Carrer de la Llum, 2, 26, 08290 Cerdanyola del Vallès, Barcelona, Spain.

[5] ICREA, Pg. Lluís Companys 23, 08010 Barcelona, Spain.

Table of Contents

Section S1. General Methods and Materials 3

S1.1 Chemicals and reagents 3

S1.2 Instruments 3

Section S2. Synthetic Procedures 5

S2.1 Synthesis of 4,4′,4″-s-triazine-2,4,6-triyl-tribenzoic acid (H_3TATB) 5

S2.2 Synthesis of (In)BCN-20B 5

S2.3 Synthesis of (Sc)BCN-20C 6

Section S3. (In)BCN-20B 7

Section S4. (In)BCN-20B' 15

Section S5. (In)BCN-20A 21

Section S6. Structural Evolution from (Sc)BCN-20C to (Sc)BCN-20A 28

References 46

Section S1. General Methods and Materials

S1.1 Chemicals and reagents

Scandium(III) nitrate hydrate and indium(III) nitrate hydrate were purchased from Alfa Aesar. 4-Carboxycinnamic acid (H_2CCA) was purchased from Biosynth. 1,3,5-Tris(4-carboxyphenyl)benzene (H_3BTB) was purchased from Chemextension. Concentrated nitric acid was purchased from Fischer Scientific. Toluene was purchased from Labkem. Acetic anhydride, chromium(VI) oxide, cyanuric chloride and NaOH were purchased from Sigma-Aldrich. Acetic acid, chloroform, *N,N*-dimethylformamide (DMF), methanol, tetrahydrofuran (THF) and 4,4'-stilbenedicarboxylic acid (H_2Sti) were purchased from TCI Chemicals. All the reagents and solvents were used as received without further purification. The deionized water from all the aqueous solutions in the article was obtained from a Milli-Q® system (18.2 MΩ·cm).

S1.2 Instruments

Single-Crystal X-Ray Diffraction (SCXRD) data of (In)BCN-20B, (In)BCN-20B', (In)BCN-20A, (Sc)BCN-20C, (Sc)BCN-20C' and (Sc)BCN-20A were collected at 100 K at XALOC beamline at ALBA synchrotron (0.82653 Å).[1] Data were indexed, integrated and scaled using the XDS program.[2] Absorption correction was not applied. The structures were solved by direct methods and subsequently refined by correction of F2 against all reflections, using SHELXT2018 within Olex2 package and WinGX (version 2021.3).[3,4] All nonhydrogen atoms were refined with anisotropic thermal parameters by full-matrix least-squares calculations on F2 using the program SHELXL2018.[5] We treated the presence of disordered solvent molecules in the cavities of all structures running solvent mask using Olex2 solvent mask or after location of the cage atoms.[6,7] Hydrogens atoms were inserted at calculated positions and constrained with isotropic thermal parameters.

Powder X-ray Diffraction (PXRD) data were recorded on an X'Pert PRO MPD analytical diffractometer (Panalytical) at 45 KV, 40 mA using CuKα radiation (λ = 1.5418 Å). Synchrotron X-ray powder diffraction patterns were collected at the BL13-XALOC beamline at the ALBA synchrotron using a capillary of 1.0 mm inner diameter. Experiments were carried out using a monochromatic x-ray beam with a wavelength of λ = 0.82653 Å. Data were collected using a PILATUS 6M DECTRIS detector. The powder diffraction patterns were radially integrated using FIT2D program.[8]

Proton Nuclear Magnetic Resonance (^{1}H NMR) spectra were collected in a Bruker

Avance NEO 400 MHz Spectrometer at "Servei de Ressonància Magnètica Nuclear" from Autonomous University of Barcelona (UAB).

Fourier Transform Infrared (FT-IR) spectra were acquired on a Bruker Tensor 27FT-IR spectrometer equipped with a Golden Gate diamond attenuated total reflection (ATR) cell. All spectra were collected neat in ambient atmosphere.

Ozonolysis was carried out using an ozone generator GHBZO3-E Commercial Ozone Generator from ZonoSistem equipped with ozone analyzer UVOZ-1200.

Electrospray Ionization Mass Spectrometry (ESI-MS) spectra were obtained in an Agilent 6210 G1969A LC/MSD TOF mass spectrometer. All the samples were measured in the negative-ionization mode.

Optical Microscopy images were obtained with a digital camera connected to a Nikon Eclipse LV100 light microscope and the software NIS-Elements F 3.0.

Section S2. Synthetic Procedures

S2.1 Synthesis of 4,4′,4′′-s-triazine-2,4,6-triyl-tribenzoic acid (H_3TATB)

H_3TATB ligand was synthesized as reported elsewhere.[9,10] H^1NMR (400 MHz, DMSO-d_6): δ 8.22 (d, 6H), 8.87 (d, 6 H), 8.64 (d, 6 H), 13.36 (s, 3H) ppm.

S2.2 Synthesis of (In)BCN-20B

Synthesis of (In)BCN-20B. A mixture of $In(NO_3)_3{\cdot}xH_2O$ (30 mg, 0.10 mmol), H_3TATB (16 mg, 0.04 mmol) and H_2CCA (9 mg, 0.05 mmol) was dispersed in 3 mL of DMF in a 23 mL scintillation vial. The procedure was followed by addition of 0.6 mL of 3.5 M HNO_3 in DMF solution. The vial was sealed and heated in an isothermal oven at 120 °C for 30 h. After cooling down to room temperature, colorless hexagon-shaped crystals suitable for SCXRD were collected by filtration and then washed with DMF (3 × 20 mL) for 3 days. Afterwards, the sample was exchanged by THF (3 × 20 mL) for another 3 days and the resulting (In)BCN-20B crystals were dried at room temperature (25 mg).

Synthesis of (In)BCN-20B’. (In)BCN-20B’ was obtained through a solid-gas ozonolysis reaction using a similar procedure previously reported by our group.[11-13] Briefly, ~25 mg of (In)BCN-20B were filtered and air-dried for 15 min and then, packed into the plastic tube and subsequently subject to the ozonator setup. The concentration of ozone was set at 15 g/Nm^3. The ozonolysis reaction was held for 35 min. Afterwards, the sample was directly collected from the plastic tube.

Synthesis of (In)BCN-20A. (In)BCN-20A was obtained by immersion of ~20 mg of (In)BCN-20B’ into 2 mL of DMF for 1 week. The sample was then exchanged with THF for three times and dried (~20 mg).

Solvent-Assisted Linker Exchange (SALE) Experiments. Note that these reactions were done to evaluate the possibility to replace directly the CCA linkers in (In)BCN-20B by BDC linkers, to form (In)BCN-20A. These reactions were done by incubating (In)BCN-20B with a DMF solution of terephthalic acid (H_2BDC), systematically changing the concentration of H_2BDC and the temperature. Briefly, 10 mg of (In)BCN-20B crystals were soaked into the DMF solution containing H_2BDC at different concentrations (1, 2, 4, 10, 20, 40 and 60 mM) under different temperatures (25 °C 65 °C and 85 °C) for 3 days. The supernatant was decanted and refreshed with the DMF

solution of H_2BDC every 12 hours. In all cases, we did not observe the transformation from (In)BCN-20B to (In)BCN-20A.

S2.3 Synthesis of (Sc)BCN-20C

Synthesis of (Sc)BCN-20C. A mixture of $Sc(NO_3)_3 \cdot xH_2O$ (21 mg, 0.08 mmol), H_3BTB (20 mg, 0.05 mmol) and H_2sti (13.6 mg, 0.05 mmol) was dispersed in 3.5 mL of DMF in a 23 mL scintillation vial. The procedure was followed by addition of 0.3 mL of 3.5 M HNO_3 aqueous solution. The vial was sealed and heated in an isothermal oven at 120 °C for 30 h. After cooling down to room temperature, colorless hexagon-shaped crystals suitable for SCXRD were collected by filtration and then washed with DMF (3 × 20 mL) for 3 days. After that, the sample was exchanged by THF (3 × 20 mL) for another 3 days, and the resulting (Sc)BCN-20C crystals were dried at room temperature (26 mg).

Synthesis of (Sc)BCN-20C'. (Sc)BCN-20C' was obtained through a solid-gas ozonolysis reaction using a similar procedure previously reported by our group.[11-13] Briefly, ~25 mg of (Sc)BCN-20C were filtered and air-dried for 15 min and then, packed into the plastic tube and subsequently subject to the ozonator setup. The concentration of ozone was set at 15 g/Nm^3. The ozonolysis reaction was held for 5 mins. Afterwards, the sample was directly collected from the plastic tube.

Synthesis of (Sc)BCN-20A. (Sc)BCN-20A was obtained by immersion of ~20 mg of (Sc)BCN-20C' into 2 mL of DMF for 1 week. The sample was then exchanged with THF for three times and dried (~20 mg).

Solvent-Assisted Linker Exchange (SALE) Experiments. Note that these reactions were done to evaluate the possibility to replace directly the CCA linkers in (Sc)BCN-20C by BDC linkers, to form (Sc)BCN-20A. These reactions were done by incubating (Sc)BCN-20C with a DMF solution of terephthalic acid (H_2BDC), systematically changing the concentration of H_2BDC and the temperature. Briefly, 10 mg of (Sc)BCN-20C crystals were soaked into the DMF solution containing H_2BDC at different concentrations (1, 2, 4, 10, 20, 40 and 60 mM) under different temperatures (25 °C 65 °C and 85 °C) for 3 days. The supernatant was decanted and refreshed with the DMF solution of H_2BDC every 12 hours. In all cases, we did not observe the transformation from (Sc)BCN-20C to (Sc)BCN-20A.

Section S3. (In)BCN-20B

Table S1. Crystal data and structure refinement for (In)BCN-20B

Identification code	(In)BCN-20B
CCDC Number	2264126
Empirical formula	$C_{135}H_{90}In_9N_{15}O_{51}$
Formula weight	3771.59
Temperature/K	100.15
Crystal system	trigonal
Space group	$R\bar{3}c$
a/Å	30.820(1)
b/Å	30.820(1)
c/Å	95.540(1)
α/°	90
β/°	90
γ/°	120
Volume/Å^3	78592.5(1)
Z	12
ρ_{calc}g/cm^3	0.956
μ/mm^{-1}	1.243
F(000)	22248.0
Crystal size/mm^3	0.1 × 0.08 × 0.08
Radiation	Synchrotron (λ = 0.82653 Å)
2Θ range for data collection/°	2.032 to 68.158
Index ranges	$-41 \leq h \leq 41$, $-37 \leq k \leq 37$, $-128 \leq l \leq 128$
Reflections collected	332592
Independent reflections	20442 [R_{int} = 0.0565, R_{sigma} = 0.0212]
Data/restraints/parameters	20442/0/652
Goodness-of-fit on F^2	1.092
Final R indexes [I>=2σ (I)]	R_1 = 0.0622, wR_2 = 0.2025
Final R indexes [all data]	R_1 = 0.0700, wR_2 = 0.2089
Largest diff. peak/hole / e Å^{-3}	1.44/-1.61

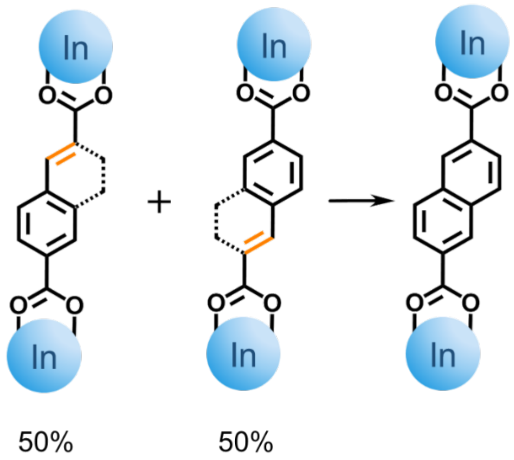

Figure S1. Representation showing that the CCA linker is symmetrically disordered about an inversion center, which results in similarity of spatial occupation between the dislocated aromatic rings of CCA and naphthalene-2,6-dicarboxylate (NDC).

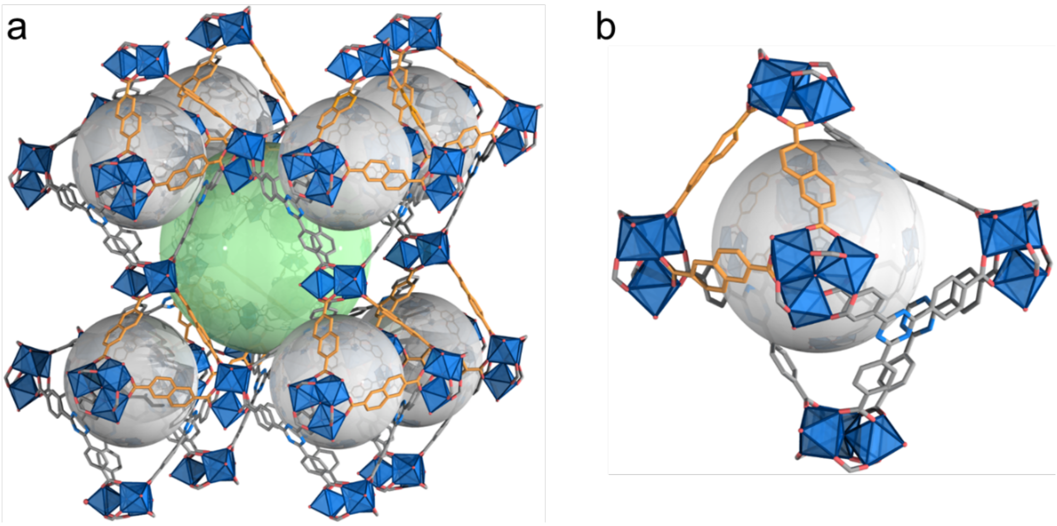

Figure S2. a) Crystal structure of (In)BCN-20B. b) Octahedral cage. Note that the disorder in the CCA linker resembles a naphthalene-2,6-dicarboxylate (NDC) linker (see also **Figure S1**).

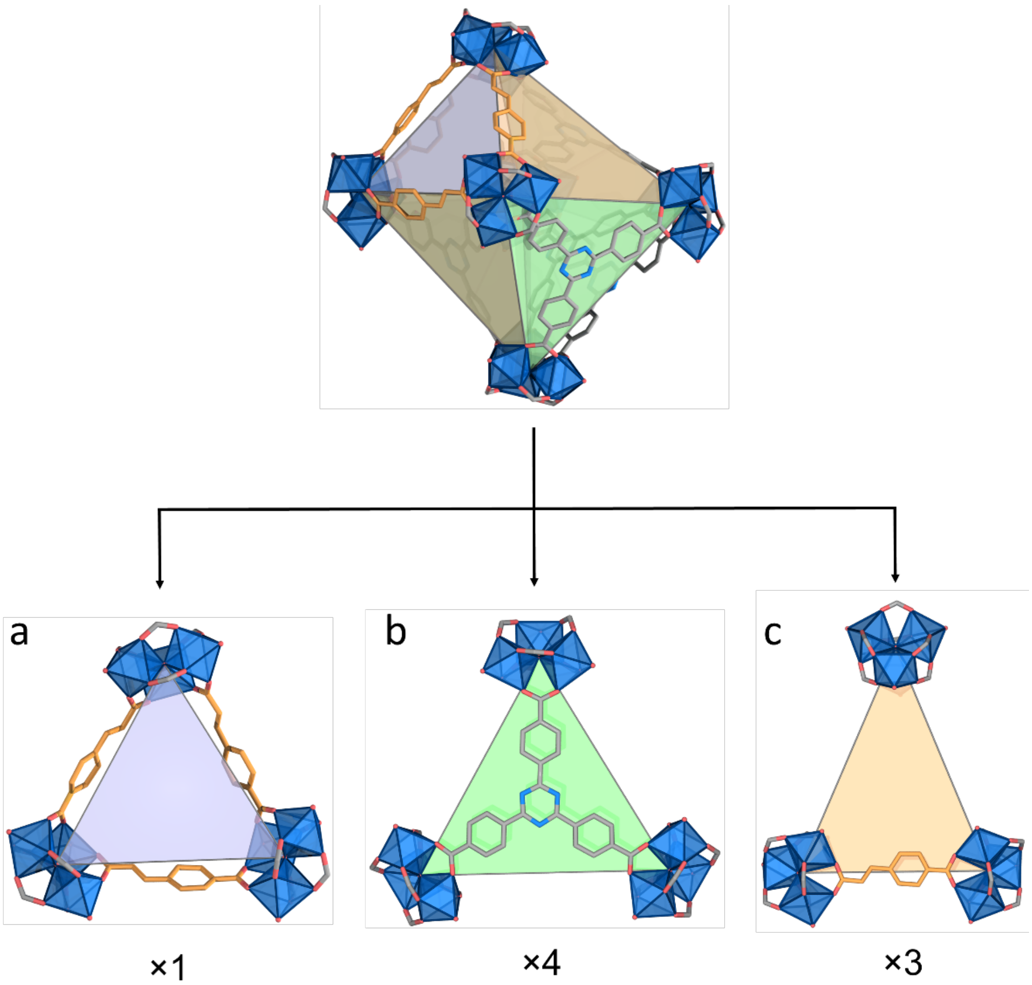

Figure S3. Representation of the three different types of triangular faces composing the octahedral cage in (In)BCN-20B.

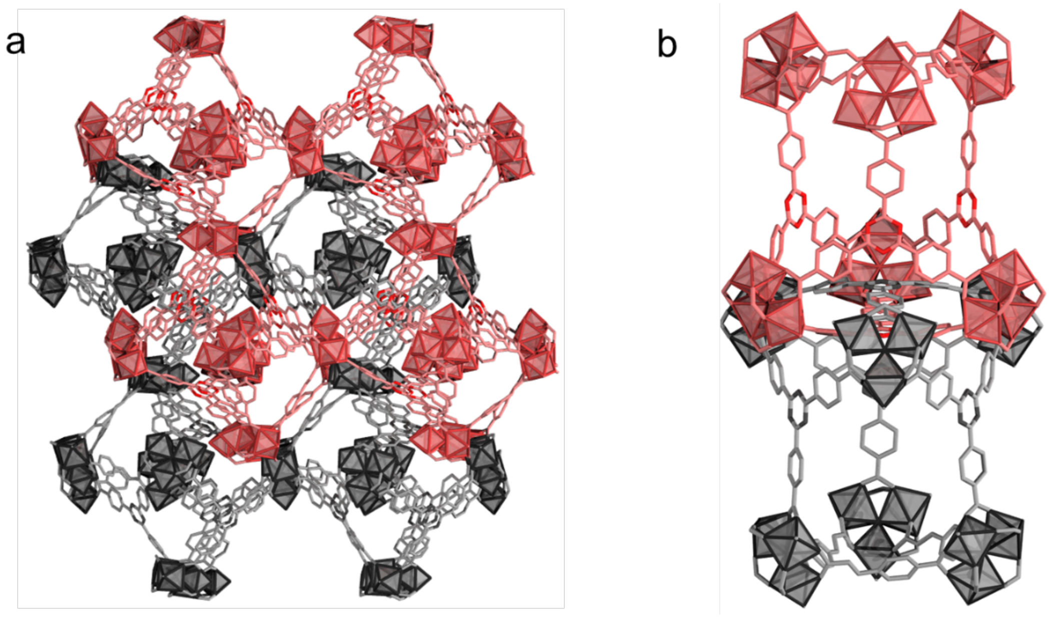

Figure S4. Crystal structure of (In)BCN-20B, showing (a) the 2-fold interpenetrated structure; and (b) two interlocked octahedral cages by interpenetration.

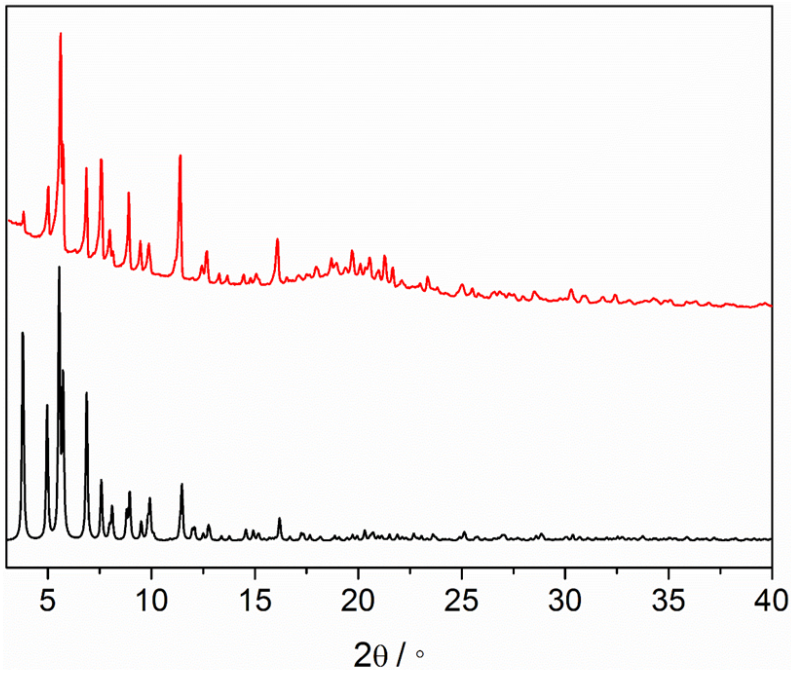

Figure S5. PXRD patterns of simulated (In)BCN-20B (black) and as-made (In)BCN-20B (red).

H[1]NMR was also used to characterize the phase purity of (In)BCN-20B. To this end, 10 mg of (In)BCN-20B was first digested using an aqueous solution (200 μL) of 5 wt% HF and heating at 120 °C overnight. Afterwards, 600 μL of DMSO-d_6 was directly added to the resulting solution. Note here that, in (In)BCN-20B, the ratio of TATB over CCA is 4/3.

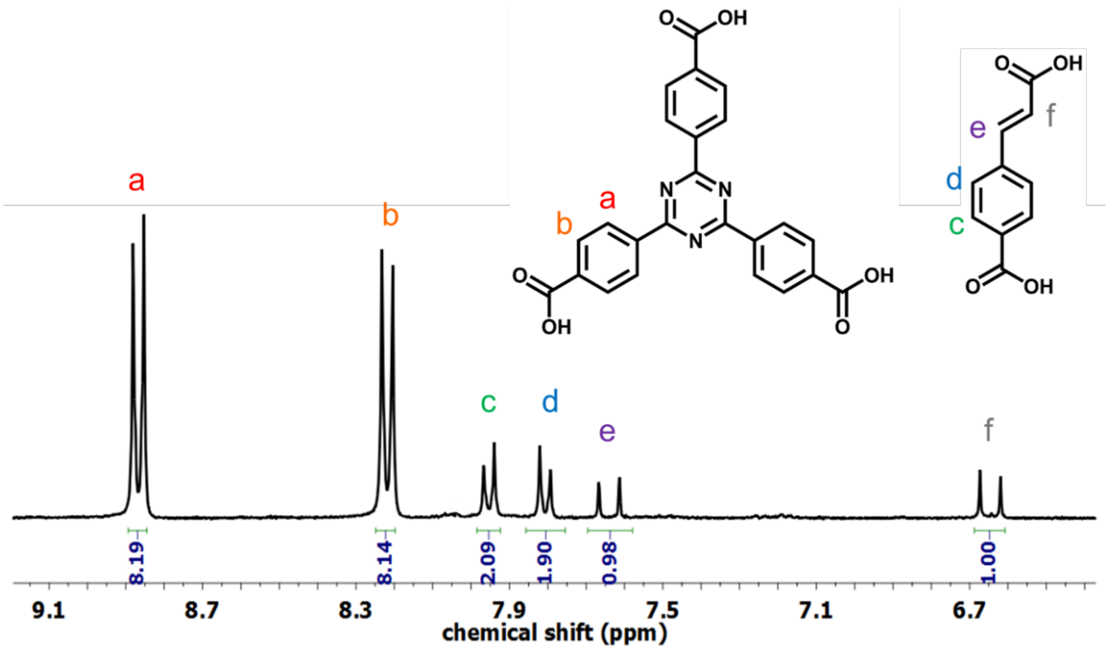

Figure S6. H[1]NMR spectrum (400 MHz) of digested (In)BCN-20B displaying the ratio between TATB and CCA. Note that the experimental ratio of TATB over CCA is 4/3, matching the expected one.

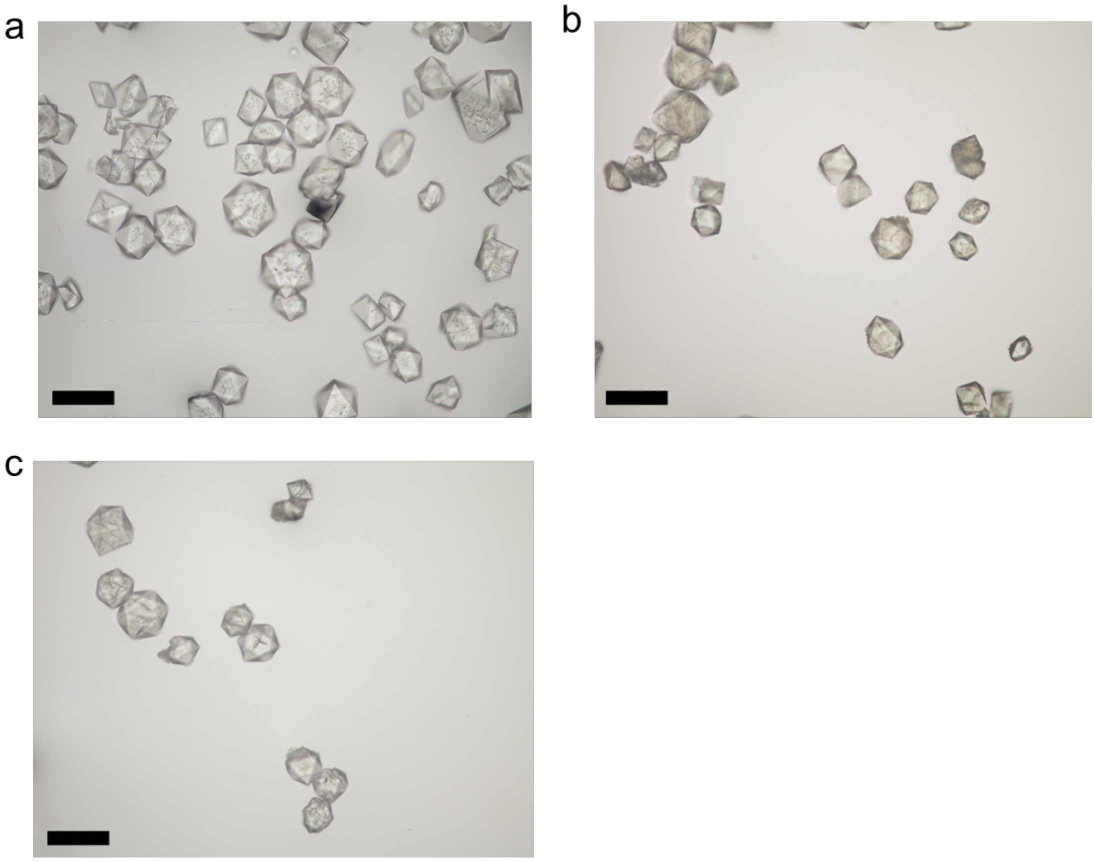

Figure S7. Comparison between the optical microscopy images of (a) (In)BCN-20B, (b) (In)BCN-20B', (c) (In)BCN-20A. Scale bars: 10 µm. Note that no apparent damage of the crystals is observed during the transformation from (In)BCN-20B to (In)BCN-20A.

Section S4. (In)BCN-20B’

Table S2. Crystal data and structure refinement for (In)BCN-20B’

Identification code	(In)BCN-20B’
CCDC Number	2264123
Empirical formula	$C_{129}H_{48}In_9N_{13.5}O_{49.5}$
Formula weight	3612.19
Temperature/K	100.15
Crystal system	trigonal
Space group	$R\bar{3}m$
a/Å	30.8299(10)
b/Å	30.8299(10)
c/Å	47.5919(20)
α/°	90
β/°	90
γ/°	120
Volume/Å^3	39175.0(22)
Z	6
ρ_{calc}g/cm^3	0.919
μ/mm^{-1}	1.243
F(000)	10521.0
Crystal size/mm^3	0.1 × 0.07 × 0.07
Radiation	Synchrotron (λ = 0.82653 Å)
2Θ range for data collection/°	3.072 to 53.382
Index ranges	$0 \leq h \leq 28$, $0 \leq k \leq 16$, $-51 \leq l \leq 51$
Reflections collected	108168
Independent reflections	6247 [R_{int} = 0.1242, R_{sigma} = 0.0535]
Data/restraints/parameters	6247/12/295
Goodness-of-fit on F^2	1.206
Final R indexes [I>=2σ (I)]	R_1 = 0.1061, wR_2 = 0.2997
Final R indexes [all data]	R_1 = 0.1279, wR_2 = 0.3275
Largest diff. peak/hole / e Å^{-3}	2.32/-0.95

Previous to this experiment, 10 mg of (In)BCN-20B' was first digested using an aqueous solution (200 μL) of 5 wt% HF and heating at 120 °C overnight. Afterwards, 600 μL of DMSO-d_6 was directly added to the resulting solution.

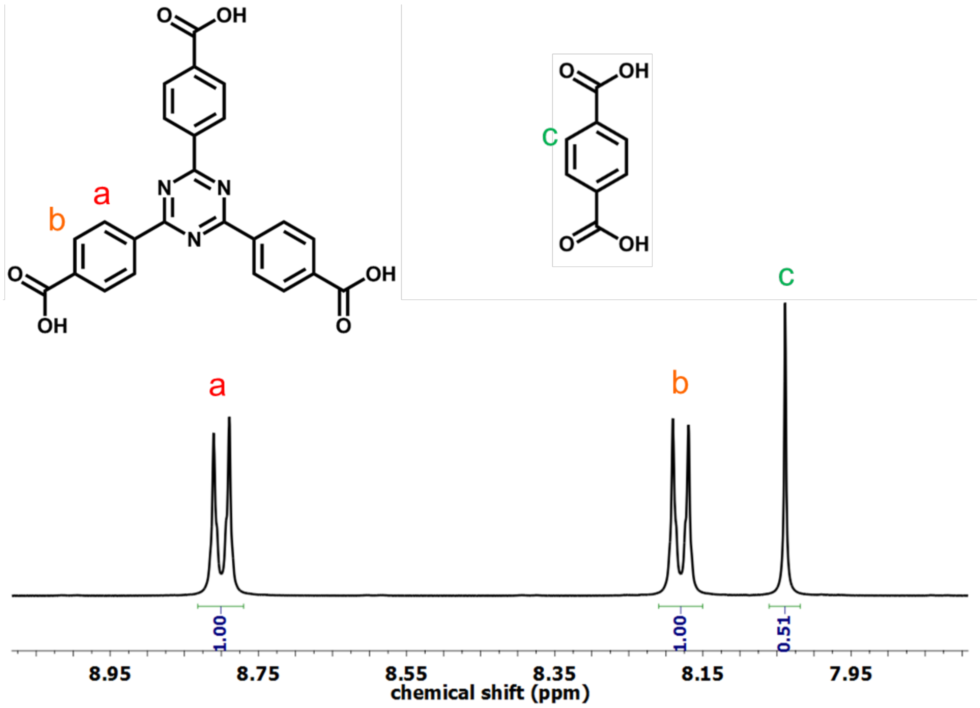

Figure S8. H^1NMR spectrum (400 MHz) of digested (In)BCN-20B' displaying the ratio between TATB and BDC. Note that the experimental ratio of TATB over BDC is 4/3, matching well with the ratio expected from the cleavage of each CCA linker into a BDC linker and a glyoxylate linker.

Previous to the ESI-MS measurements, 10 mg of (In)BCN-20B' was digested using an aqueous solution (200 μL) of 5 wt% HF at room temperature. Then, 400 μL of DMSO-d_6 was directly added to make a clear solution.

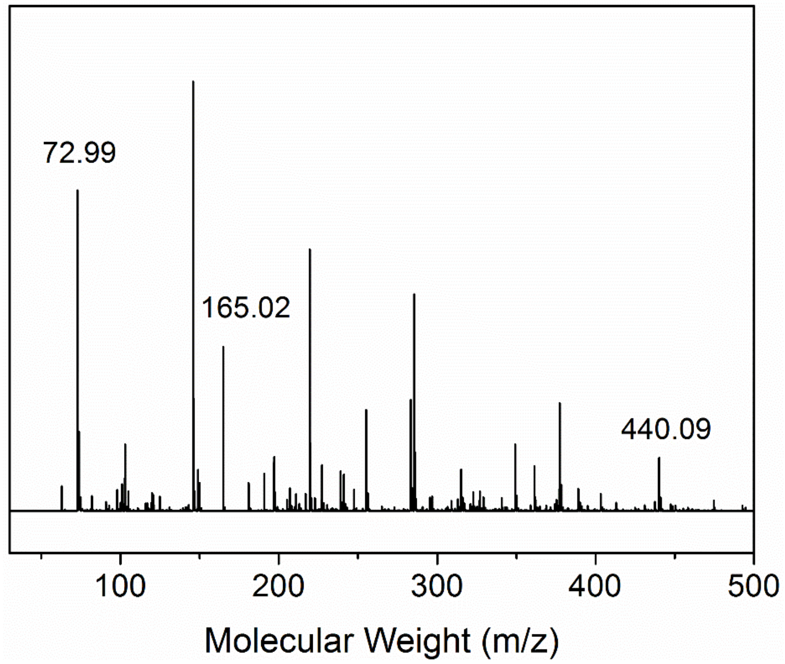

Figure S9. Negative mode ESI-MS spectrum of digested (In)BCN-20B'. Note that the peak at m/z = 72.99 is assigned to the glyoxylic acid, corresponding to the formula $[C_2H_2O_3\text{-}H]^-$: expected = 73.03, found = 72.99. The peak at m/z = 165.02 is assigned to BDC, corresponding to the formula $[C_8H_6O_4\text{-}H]^-$: expected = 165.03, found = 165.02. The peak at m/z = 440.09 is assigned to H_3TATB, corresponding to the formula $[C_{24}H_{15}N_3O_6\text{-}H]^-$: expected = 440.10, found = 440.09.

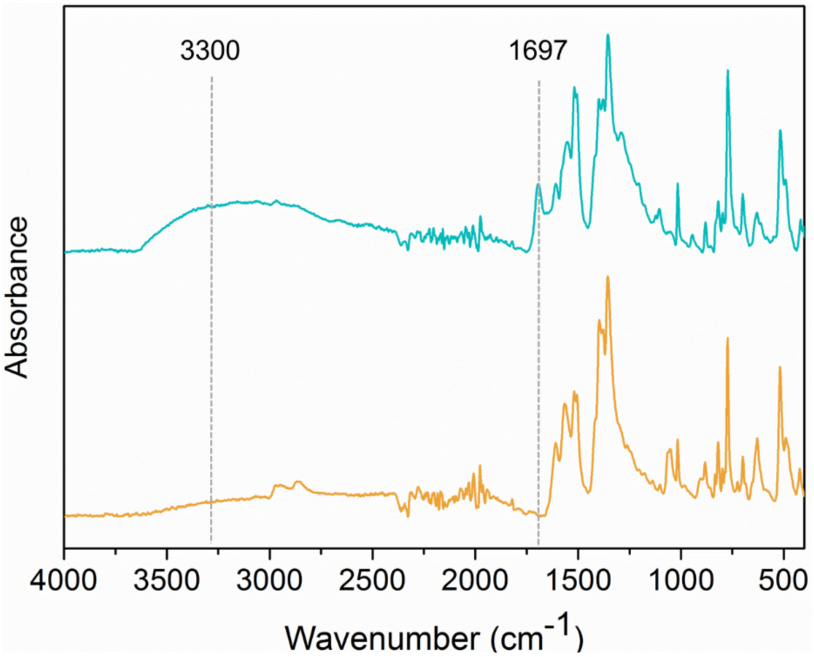

Figure S10. Full widths of FT-IR spectra of (In)BCN-20B (orange) and (In)BCN-20B' (cyan). Note that the ozonated (In)BCN-20B' exhibited a more intense C=O stretch band at 1697 cm^{-1} relative to that in the spectrum of (In)BCN-20B. This is due to the oxidative cleavage of the alkene bond of CCA linkers into a carboxylic acid group (to form a single deprotonated BDC) and an aldehyde group (to form glyoxylate) An enhanced broad band centered at 3300 cm^{-1} was observed due to the large perturbations caused by H-bonded hydroxyl groups of the free carboxylic acid groups.

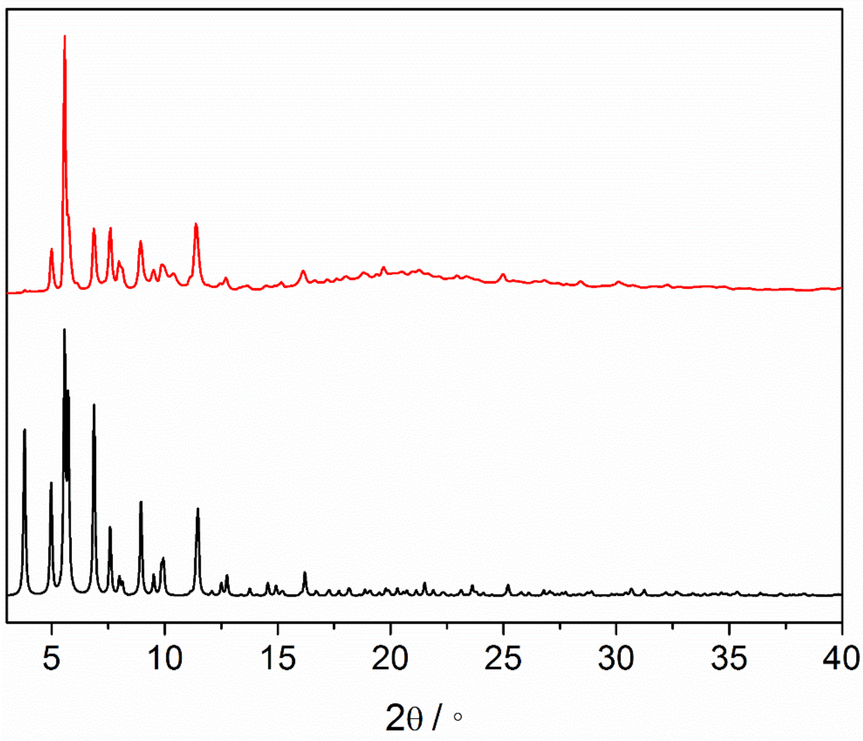

Figure S11. PXRD patterns of simulated (In)BCN-20B' (black) and as-made (In)BCN-20B' (red).

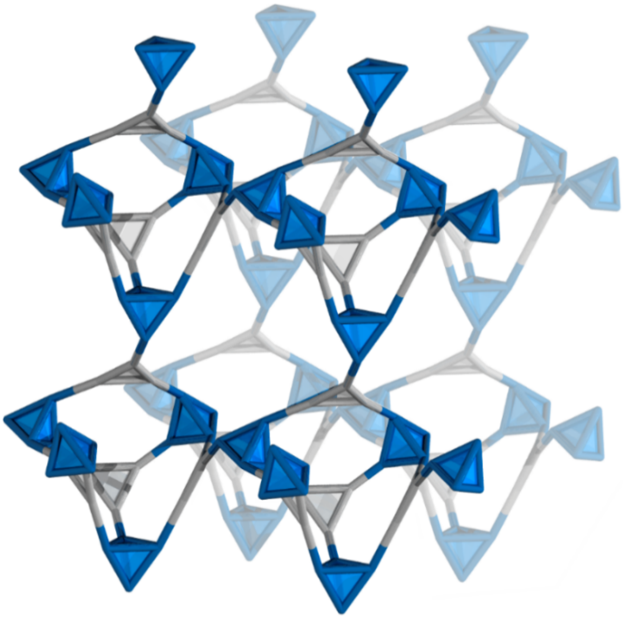

Figure S12. Representation showing the augmented net of (In)BCN-20B', containing two kinds of nodes: 4-c tetrahedral and 3-c triangular nodes.

Section S5. (In)BCN-20A

Table S3. Crystal data and structure refinement for (In)BCN-20A

Identification code	(In)BCN-20A
CCDC Number	2264121
Empirical formula	$C_{120}H_{60}In_9N_{12}O_{48}$
Formula weight	3471.18
Temperature/K	100
Crystal system	trigonal
Space group	$R\bar{3}m$
a/Å	28.7831(5)
b/Å	28.7831(5)
c/Å	47.7075(15)
α/°	90
β/°	90
γ/°	120
Volume/Å^3	34228.9(11)
Z	6
ρ_{calc}g/cm^3	1.010
μ/mm^{-1}	1.419
F(000)	10134.0
Crystal size/mm^3	0.11 × 0.09 × 0.09
Radiation	Synchrotron (λ = 0.82653 Å)
2Θ range for data collection/°	2.748 to 54.628
Index ranges	0 ≤ h ≤ 27, 0 ≤ k ≤ 15, -52 ≤ l ≤ 52
Reflections collected	99893
Independent reflections	5836 [R_{int} = 0.1013, R_{sigma} = 0.0450]
Data/restraints/parameters	5836/0/304
Goodness-of-fit on F^2	1.161
Final R indexes [I>=2σ (I)]	R_1 = 0.1052, wR_2 = 0.2921
Final R indexes [all data]	R_1 = 0.1232, wR_2 = 0.3116
Largest diff. peak/hole / e Å^{-3}	1.99/-0.62

In order to study the phase transition from (In)BCN-20B' to (In)BCN-20A, (In)BCN-20B' was incubated in DMF for 1 week and then, the dispersion was centrifuged to separate the supernatant from the solid. This DMF supernatant was analyzed by ESI-MS in a negative mode.

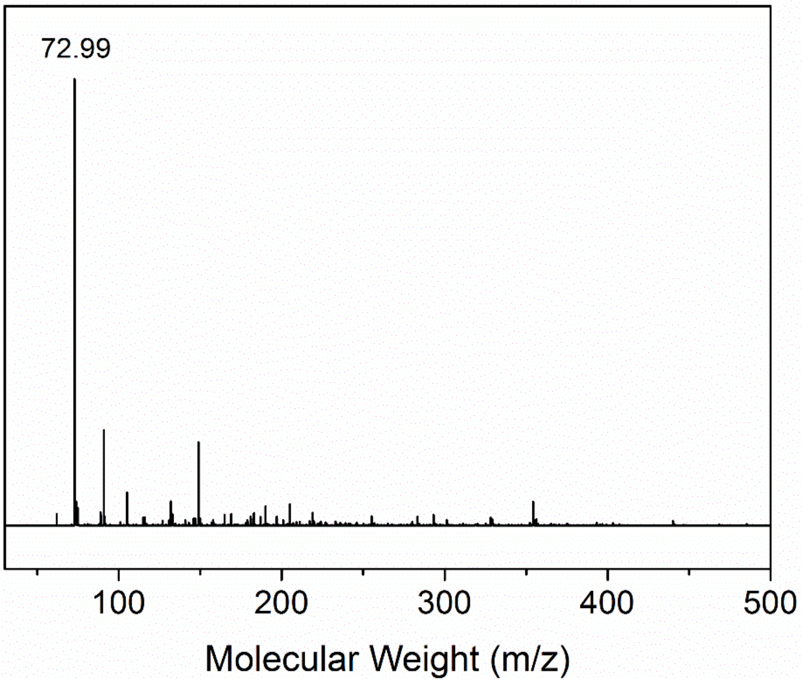

Figure S13. Negative mode ESI-MS spectrum of DMF supernatant after incubation of (In)BCN-20B' in DMF for 1 week. Note that the peak at m/z = 72.99 is assigned to the glyoxylic acid, corresponding to the formula $[C_2H_2O_3\text{-}H]^-$: expected = 73.03, found = 72.99. This result confirms that glyoxylic acid is release from (In)BCN-20B' when it is incubated in DMF.

Comparison of Unit Cells in the transformation from (In)BCN-20B to (In)BCN-20A

(In)BCN-20B crystallized in the centrosymmetric trigonal space group $R\bar{3}c$ (No. 167). However, both (In)BCN-20B' and (In)BCN-20A crystallized in trigonal Bravais lattices with the space group $R\bar{3}m$ (No. 166). The structural relationship correlates to a group-subgroup relationship of the space groups. $R\bar{3}m$ has a maximal non-isomorphic subgroup $R\bar{3}c$ (a' = −a, b' = −b, c' = 2c) with *klassengleich* transition of index 2, indicating $R\bar{3}c$ possesses a two times larger unit cell. In order to reduce the symmetry of $R\bar{3}m$ for clear comparison, the *c* parameter in (In)BCN-20B' and (In)BCN-20A is shown as $2c$ and the volume as $2V$. For the valid crystallographic information from (In)BCN-20B to (In)BCN-20A, the single crystal X-ray crystallographic data can be seen in **Tables S1-S3**.

Table S4. Comparison of the unit cell parameters of (In)BCN-20B, (In)BCN-20B', and (In)BCN-20A.

	a (Å)	*c* (Å)	*V* (Å^3)
(In)BCN-20B	30.820	95.540	78592.5
(In)BCN-20B'	30.830	95.184	78350.0
(In)BCN-20A	28.783	95.415	68457.8

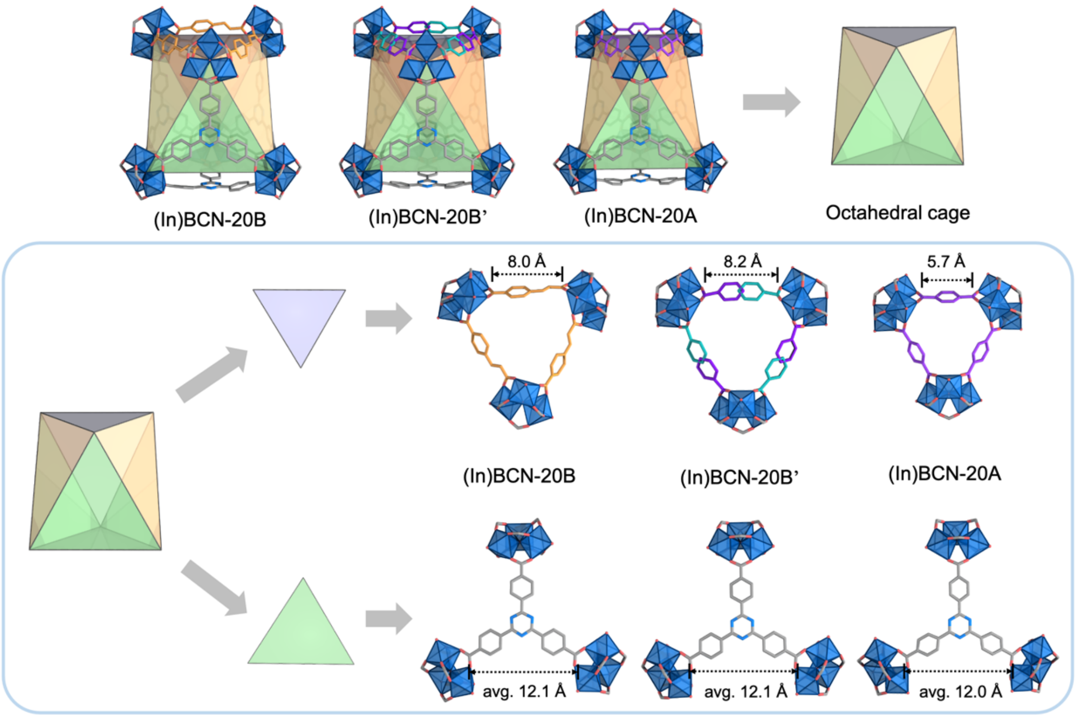

Figure S14. Schematic representation showing the single-crystal-to-single-crystal evolution from (In)BCN-20B to (In)BCN-20B’ to (In)BCN-20A, highlighting the changes in the octahedral cage.

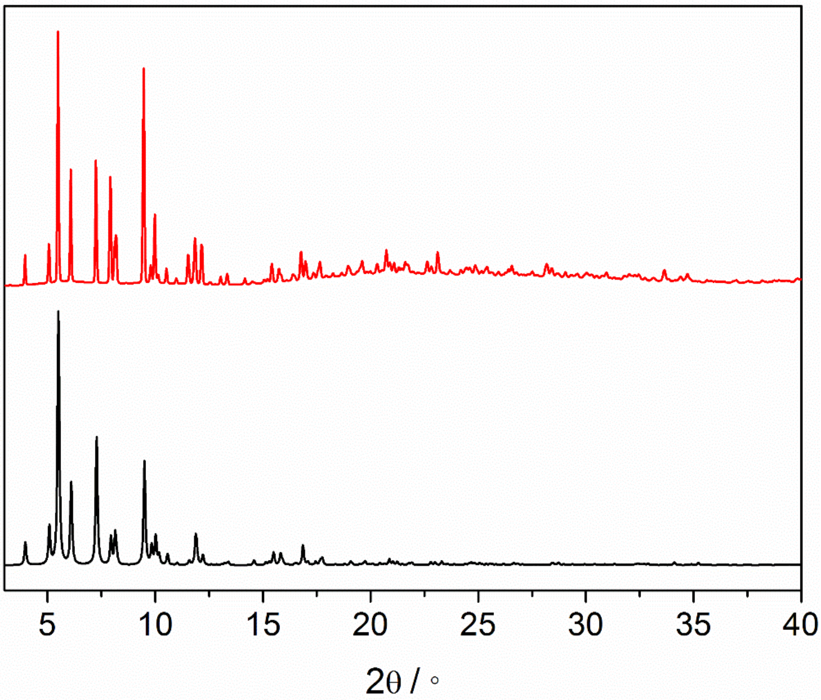

Figure S15. PXRD patterns of simulated (In)BCN-20A (black) and as-made (In)BCN-20A (red).

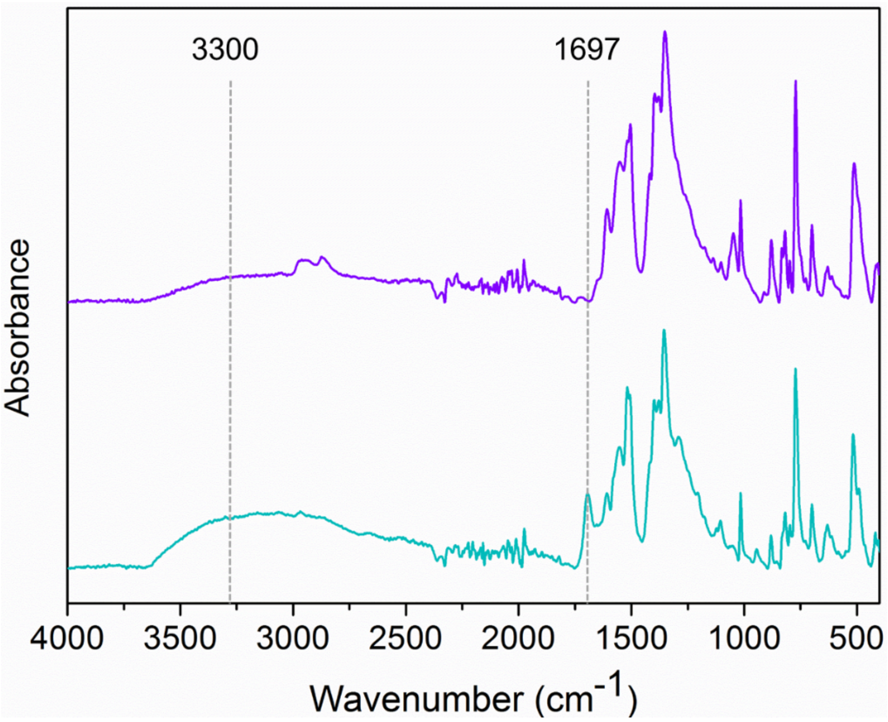

Figure S16. Full widths of FT-IR spectra of (In)BCN-20B' (cyan) and (In)BCN-20A (violet). Note that the C=O stretch band at 1697 cm^{-1} disappeared and the broad band centered at 3300 cm^{-1} was attenuated in (In)BCN-20A. This phenomenon can be ascribed to the re-coordination between In^{3+}-cluster and the free carboxylate from BDC.

Previous to this experiment, 10 mg of (In)BCN-20B' was first digested using an aqueous solution (200 µL) of 5 wt% HF and heating at 120 °C overnight. Afterwards, 600 µL of DMSO-d_6 was added to the solution.

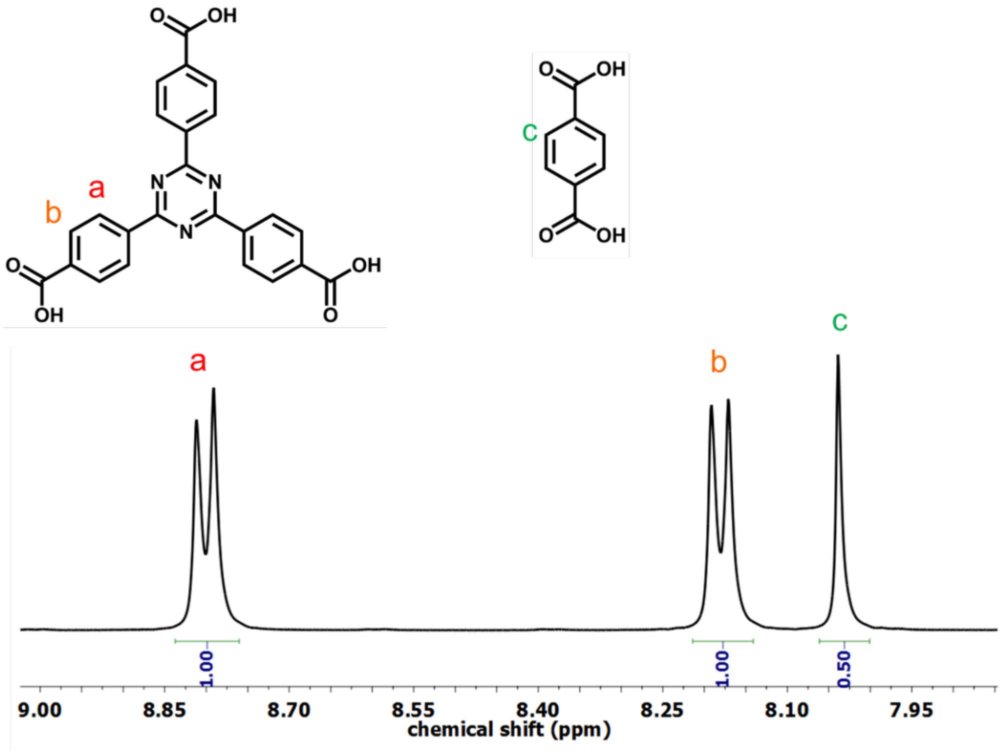

Figure S17. H^1NMR spectrum (400 MHz) of digested (In)BCN-20A displaying the ratio between TATB and BDC. Note that the experimental ratio of TATB over BDC is 4/3, matching well with the expected one and further confirming the pure phase of (In)BCN-20A.

Section S6. Structural Evolution from (Sc)BCN-20C to (Sc)BCN-20A

Table S5. Crystal data and structure refinement for (Sc)BCN-20C

Identification code	(Sc)BCN-20C
CCDC Number	2264124
Empirical formula	$C_{325}H_{178}N_3O_{99}Sc_{18}$
Formula weight	6517.97
Temperature/K	100.00
Crystal system	monoclinic
Space group	C2/c
a/Å	60.2793(3)
b/Å	34.7226(2)
c/Å	37.1357(1)
α/°	90
β/°	123.5062(3)
γ/°	90
Volume/Å^3	64811.2
Z	4
ρ_{calc}g/cm^3	0.668
μ/mm^{-1}	0.329
F(000)	13276.0
Crystal size/mm^3	0.07 × 0.06 × 0.06
Radiation	Synchrotron (λ = 0.82653 Å)
2Θ range for data collection/°	1.658 to 67.832
Index ranges	$-74 \leq h \leq 62, 0 \leq k \leq 39, 0 \leq l \leq 45$
Reflections collected	399678
Independent reflections	69415 [R_{int} = 0.0717, R_{sigma} = 0.0580]
Data/restraints/parameters	69415/0/1996
Goodness-of-fit on F^2	1.072
Final R indexes [I>=2σ (I)]	R_1 = 0.0726, wR_2 = 0.2328
Final R indexes [all data]	R_1 = 0.0850, wR_2 = 0.2419
Largest diff. peak/hole / e Å^{-3}	1.72/-0.92

Table S6. Crystal data and structure refinement for (Sc)BCN-20C'

Identification code	(Sc)BCN-20C'
CCDC Number	2264122
Empirical formula	$C_{164}H_{92}O_{48}Sc_9$
Formula weight	3235.01
Temperature/K	100.00
Crystal system	monoclinic
Space group	C2/m
a/Å	37.5283(2)
b/Å	35.3742(3)
c/Å	24.7609(2)
α/°	90
β/°	94.2397(5)
γ/°	90
Volume/Å^3	32780.9(3)
Z	4
ρ_{calc}g/cm^3	0.655
μ/mm^{-1}	0.324
F(000)	6596.0
Crystal size/mm^3	0.08 × 0.07 × 0.06
Radiation	Synchrotron (λ = 0.82653 Å)
2Θ range for data collection/°	1.918 to 62.21
Index ranges	-46 ≤ h ≤ 46, 0 ≤ k ≤ 43, 0 ≤ l ≤ 30
Reflections collected	189834
Independent reflections	33389 [R_{int} = 0.0602, R_{sigma} = 0.0453]
Data/restraints/parameters	33389/0/914
Goodness-of-fit on F^2	1.100
Final R indexes [I>=2σ (I)]	R_1 = 0.0924, wR_2 = 0.2906
Final R indexes [all data]	R_1 = 0.1169, wR_2 = 0.3167
Largest diff. peak/hole / e Å^{-3}	1.26/-0.61

Table S7. Crystal data and structure refinement for (Sc)BCN-20A

Identification code	(Sc)BCN-20A
CCDC Number	2264125
Empirical formula	$C_{135}O_{48}Sc_9$
Formula weight	2793.99
Temperature/K	100.00
Crystal system	trigonal
Space group	$R\bar{3}c$
a/Å	28.6819(8)
b/Å	28.6819(8)
c/Å	95.6062(43)
α/°	90
β/°	90
γ/°	120
Volume/Å^3	68113.4(33)
Z	12
ρ_{calc}g/cm^3	0.817
μ/mm^{-1}	0.459
F(000)	16596.0
Crystal size/mm^3	0.06 × 0.06 × 0.06
Radiation	Synchrotron (λ = 0.82635 Å)
2Θ range for data collection/°	2.75 to 32.202
Index ranges	$0 \leq h \leq 16$, $0 \leq k \leq 9$, $-63 \leq l \leq 64$
Reflections collected	45160
Independent reflections	2406 [R_{int} = 0.1354, R_{sigma} = 0.1093]
Data/restraints/parameters	2406/73/331
Goodness-of-fit on F^2	1.437
Final R indexes [I>=2σ (I)]	R_1 = 0.1343, wR_2 = 0.3641
Final R indexes [all data]	R_1 = 0.1654, wR_2 = 0.3940
Largest diff. peak/hole / e Å^{-3}	0.42/-0.31

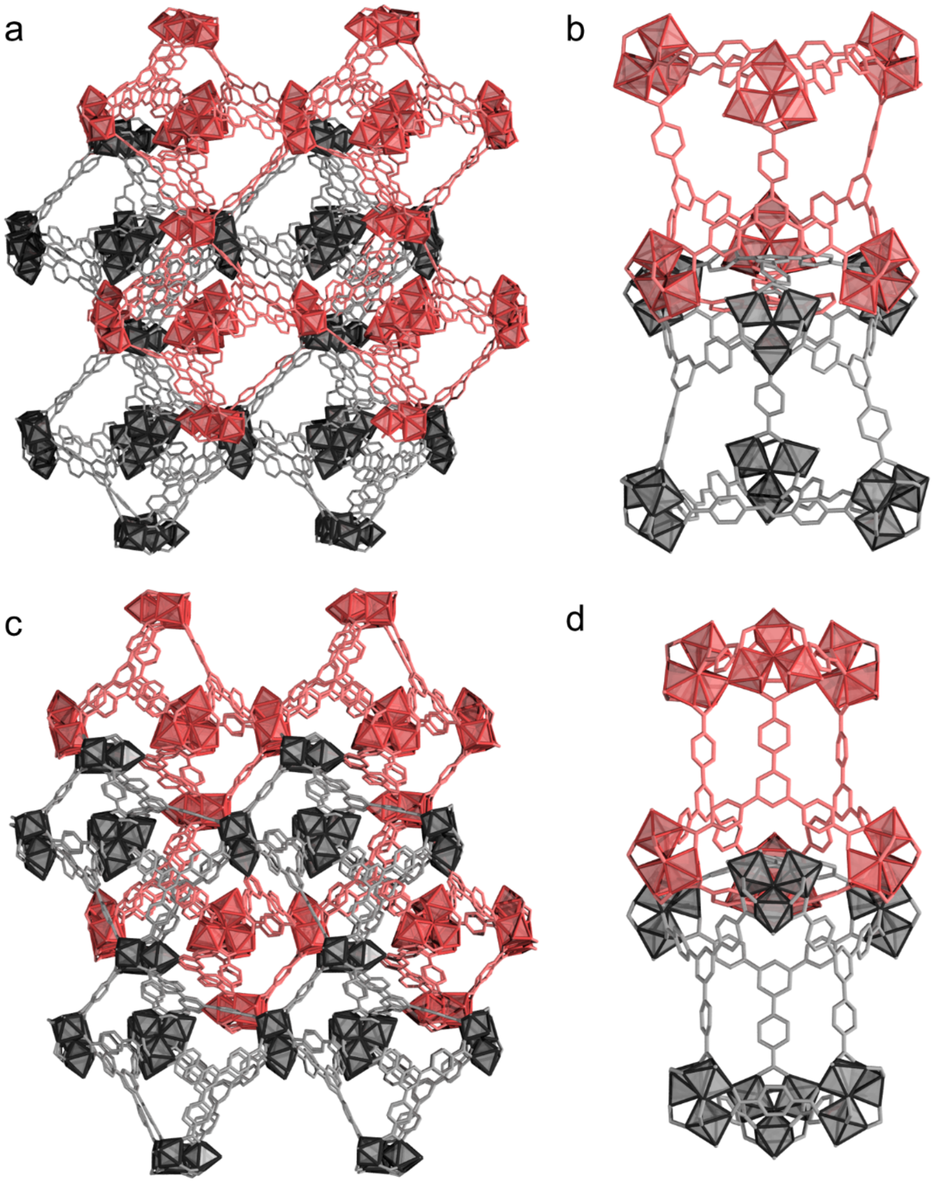

Figure S18. Comparison between the crystal structures of (Sc)BCN-20C (a,b) and (Sc)BCN-20A (c,d), showing the (a,c) 2-fold interpenetrated structures and (b,d) interlocked octahedral cages by interpenetration.

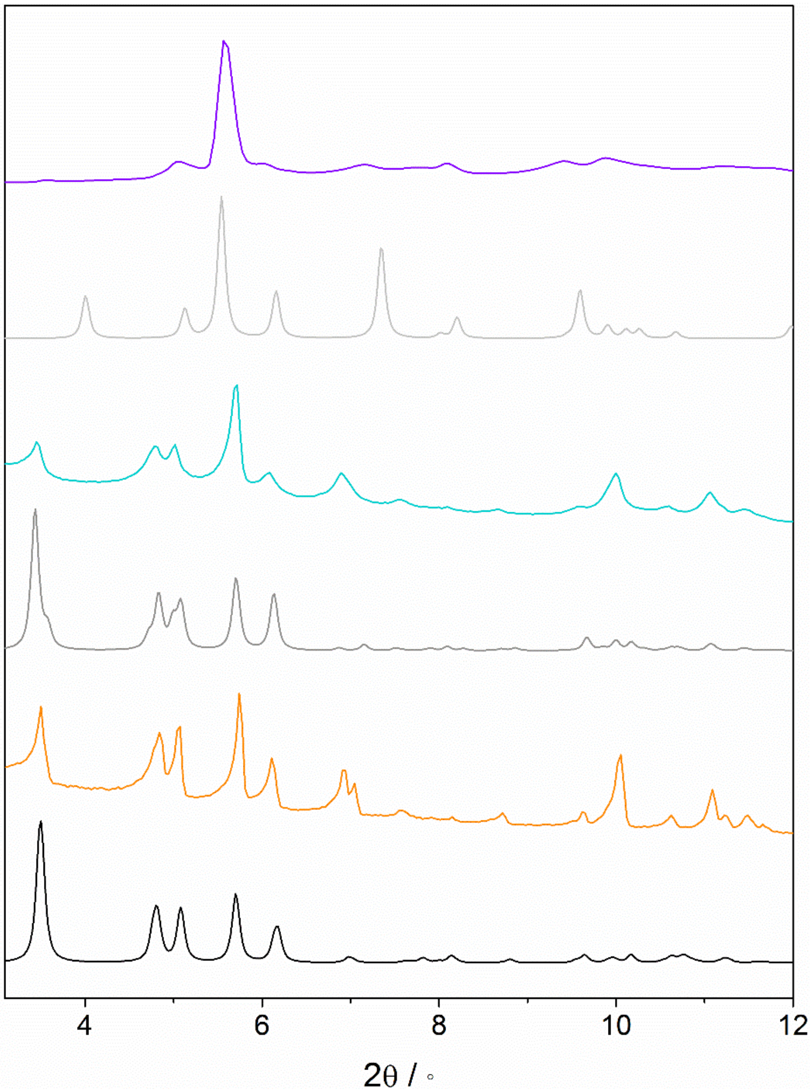

Figure S19. PXRD spectra of simulated (black) and experimental (orange) (Sc)BCN-20C; simulated (grey) and experimental (cyan) (Sc)BCN-20C'; and simulated (light grey) and experimental (violet) (Sc)BCN-20A.

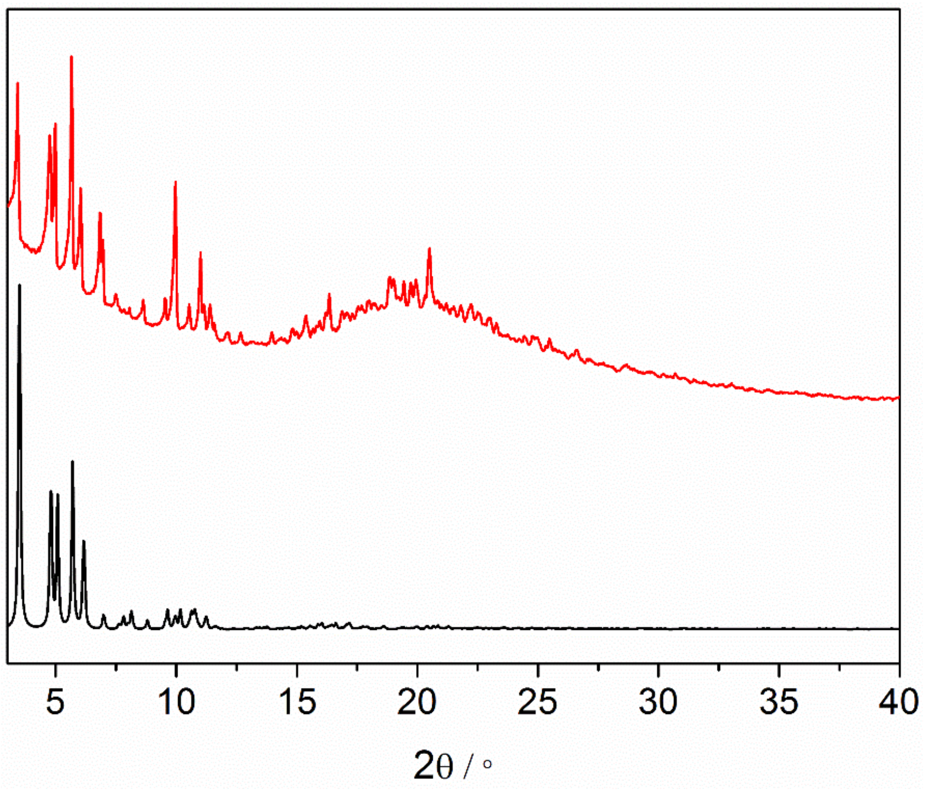

Figure S20. PXRD of simulated (Sc)BCN-20C (black) and as-made (Sc)BCN-20C (red).

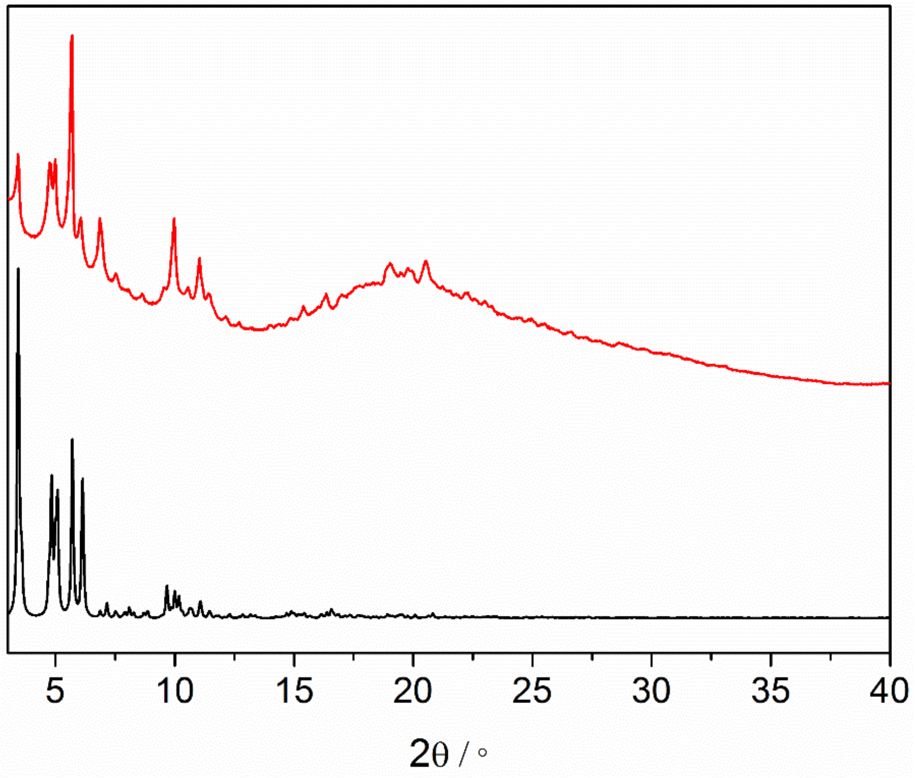

Figure S21. PXRD of simulated (Sc)BCN-20C' (black) and as-made (Sc)BCN-20C' (red).

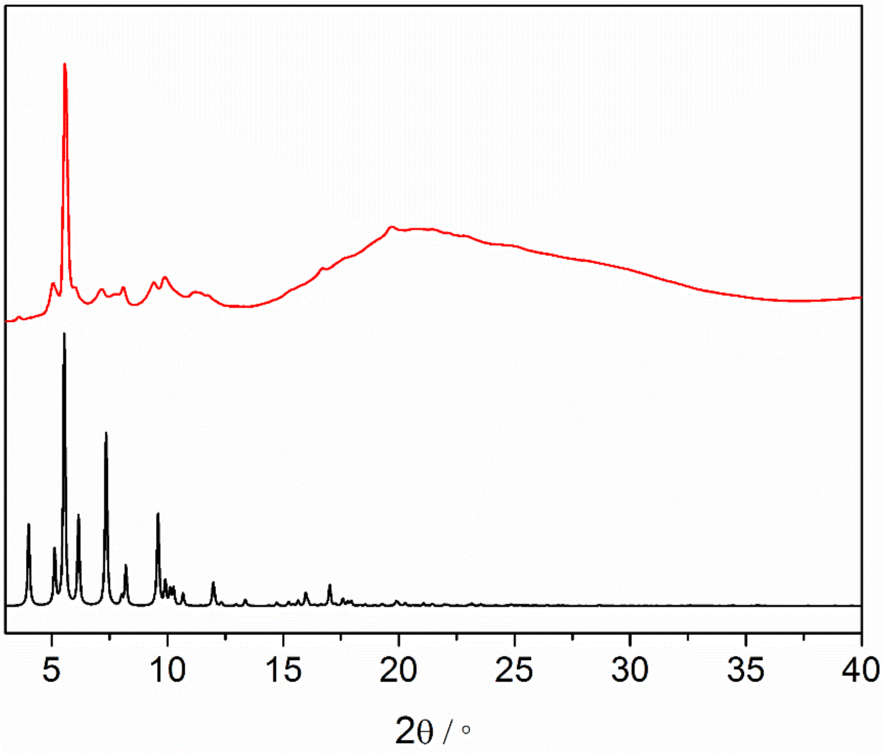

Figure S22. PXRD of simulated (Sc)BCN-20A (black) and as-made (Sc)BCN-20A (red).

H[1]NMR was also used to characterize the phase purity of (Sc)BCN-20C. To this end, 10 mg of (Sc)BCN-20C was first digested using an aqueous solution (200 μL) of 5 wt% HF and heating at 120 °C overnight. Afterwards, 600 μL of DMSO-d_6 was directly added to this solution. Note here that, in (Sc)BCN-20C, the ratio of BTB over Sti is 4/3.

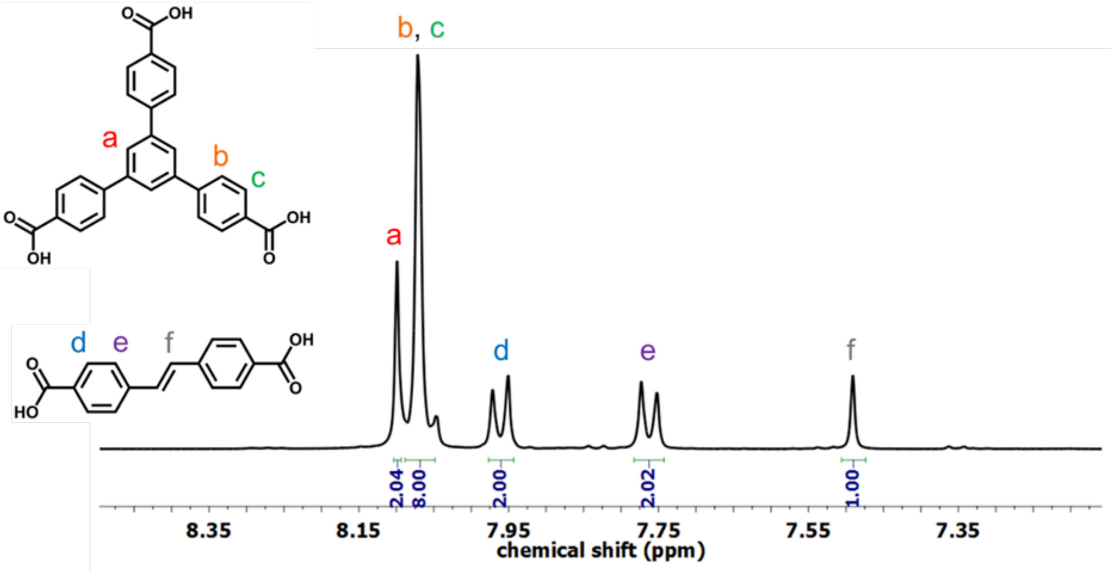

Figure S23. H[1]NMR spectrum (400 MHz) of digested (Sc)BCN-20C displaying the ratio between BTB and Sti. Note that the experimental ratio of BTB over Sti is 4/3, matching well with the expected one.

Previous to this experiment, 10 mg of (Sc)BCN-20C' was first digested using an aqueous solution (200 μL) of 5 wt% HF and heating at 120 °C overnight. Afterwards, 600 μL of DMSO-d_6 was directly added.

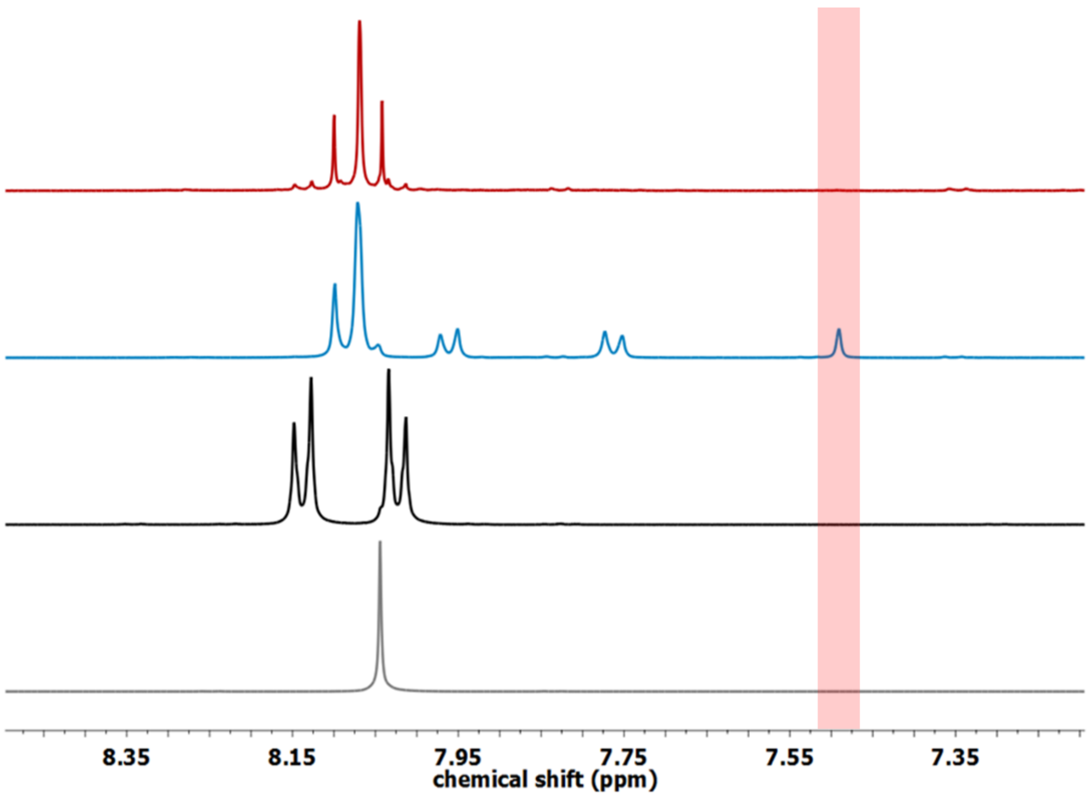

Figure S24. H^1NMR spectrum (400 MHz) of BDC (gray), 4-FBA (black), digested (Sc)BCN-20C (blue) and digested (Sc)BCN-20C' (red). Note here that the spectrum of the digested (Sc)BCN-20C' corroborated the disappearance of the characteristic peak of the olefinic protons of Sti highlighted in red at δ = 7.49 ppm, confirming the quantitative cleavage of the Sti linkers.

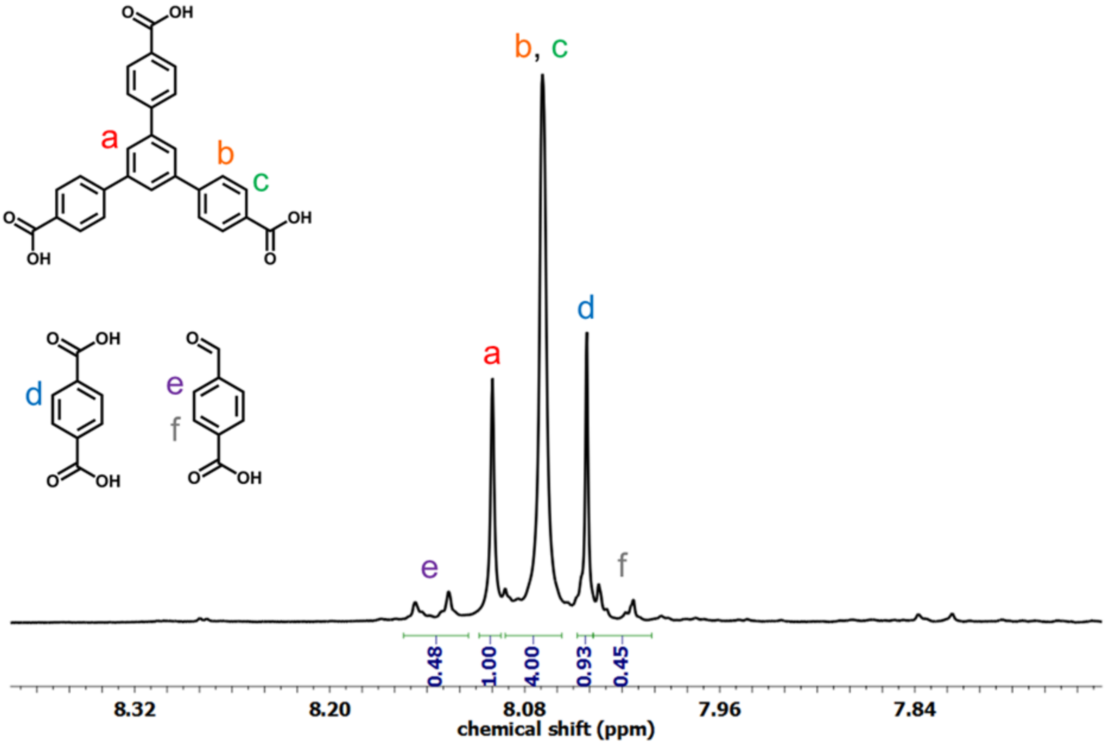

Figure S25. Enlarged H^1NMR spectrum (400 MHz) of digested (Sc)BCN-20C' displaying the ratio among BTB and ozonized products, BDC and 4-FBA. Note that the experimental ratio of BTB over BDC or 4-FBA is 4/3, matching well with the ratio expected from the cleavage of each CCA linker into a BDC ligand and a 4-FBA ligand.

Previous to this experiment, 10 mg of (Sc)BCN-20A was first digested using an aqueous solution (200 μL) of 5 wt% HF and heating at 120 °C overnight. Afterwards, 600 μL of DMSO-d_6 was directly added.

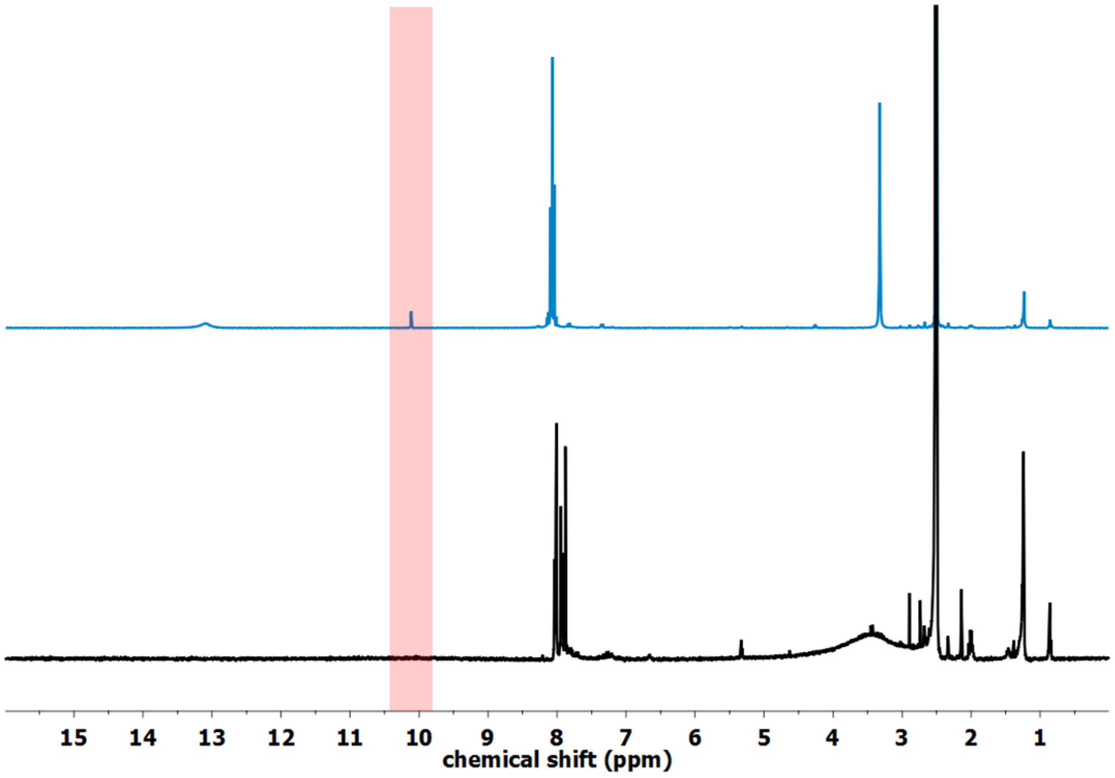

Figure S26. H^1NMR spectrum (400 MHz, DMSO-d_6) of the digested (Sc)BCN-20A (black) and (Sc)BCN-20C' (blue). Note that the proton signal of aldehyde groups (δ = 10.11 ppm) highlighted in red disappeared in (Sc)BCN-20A.

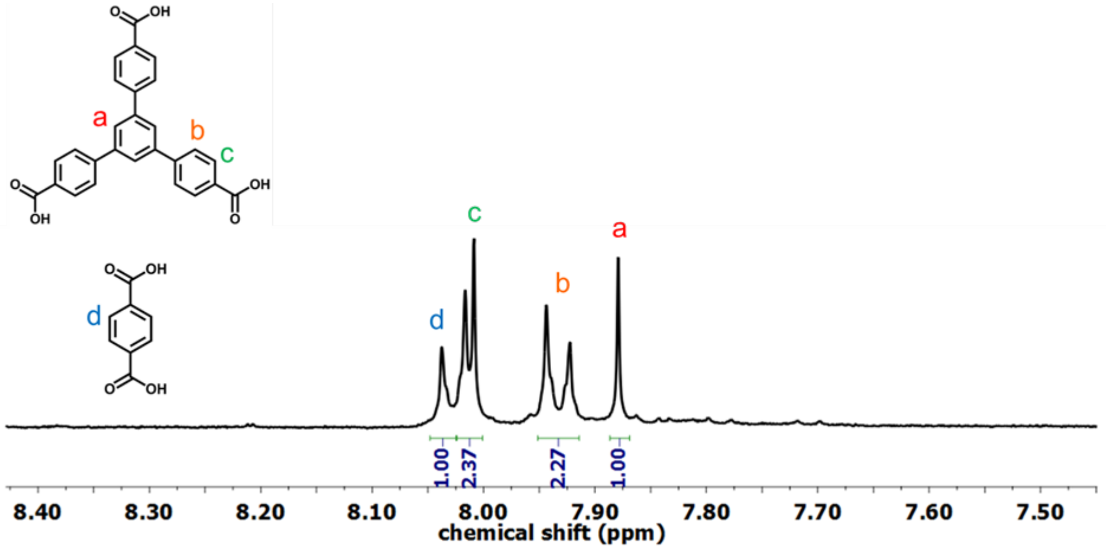

Figure S27. Enlarged H[1]NMR spectrum (400 MHz, DMSO-d_6) of the digested (Sc)BCN-20A. Note that the experimental ratio of BTB over BDC is 4/3, matching well with the expected value from (Sc)BCN-20A. Also note that the peaks corresponding to the 4-FBA have been disappeared, confirming the removal of this linker during the transformation from (Sc)BCN-20C' to (Sc)BCN-20A. Finally, the chemical shifts of protons from BTB and BDC are slightly different from those of digested (Sc)BCN-20C and (Sc)BCN-20C', which is attributed to the different acidity atmosphere.

In order to study the phase transition from (Sc)BCN-20C' to (Sc)BCN-20A, (Sc)BCN-20C' was incubated in DMF for 1 week and then, the dispersion was centrifuged to separate the supernatant from the solid. This DMF supernatant was analyzed by ESI-MS in a negative mode.

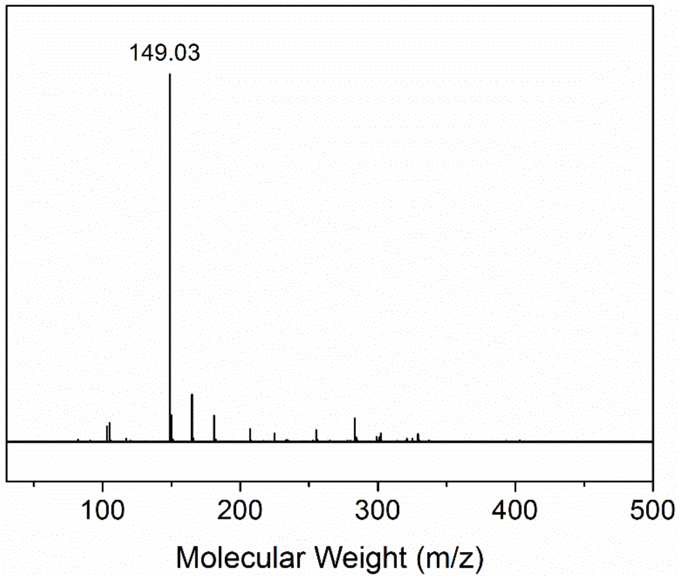

Figure S28. Negative mode ESI-MS spectrum of DMF supernatant after incubation of (In)BCN-20C' in DMF for 1 week. Note that the peak at m/z = 149.03 is assigned to 4-FBA, corresponding to the formula [$C_8H_6O_3$-H]$^-$: expected = 149.04, found = 149.03. This result further confirms that 4-FBA is released from (Sc)BCN-20C' when it is incubated in DMF.

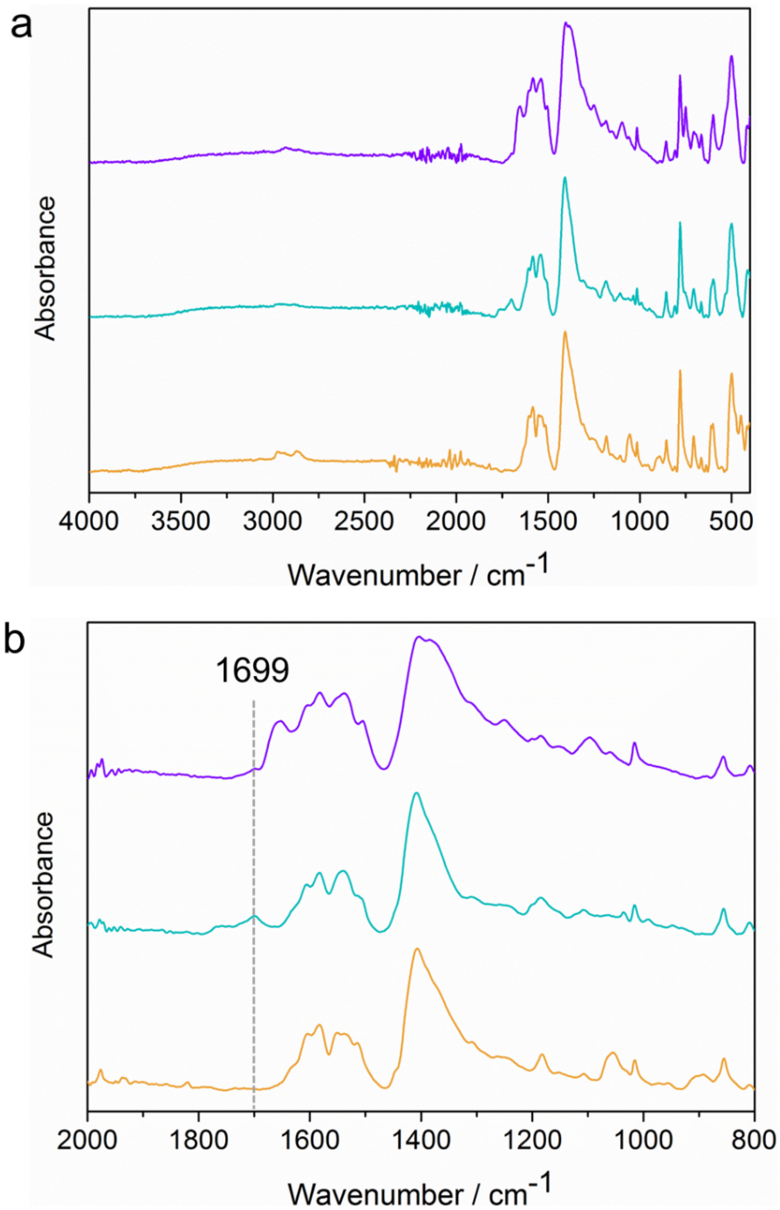

Figure S29. FT-IR spectra of (Sc)BCN-20C (orange), (Sc)BCN-20C’ (cyan) and (Sc)BCN-20A (violet). Note that the ozonated (Sc)BCN-20C’ exhibits a more intense C=O stretch band at 1699 cm^{-1} relative to those of (Sc)BCN-20C and (Sc)BCN-20A. This is due to the cleavage of Sti linkers into the BDC and 4-FBA ligands.

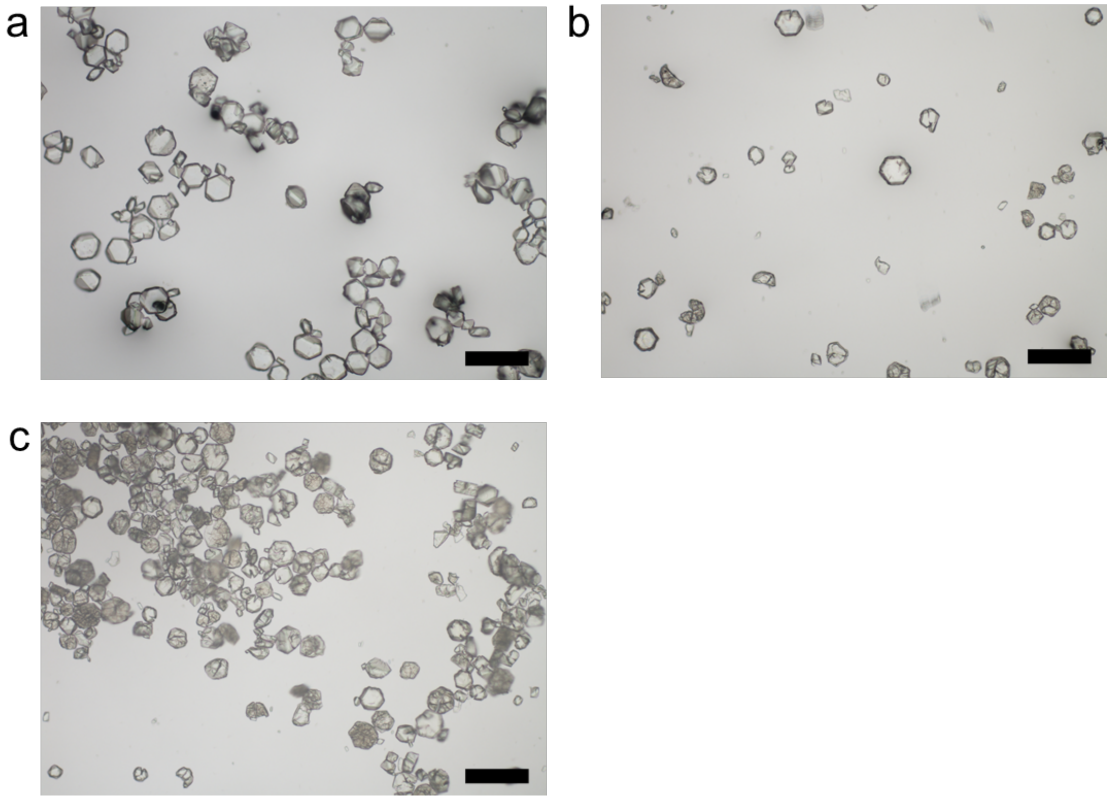

Figure S30. Comparison between the optical microscopy images of (a) (Sc)BCN-20C, (b) (Sc)BCN-20C’, (c) (Sc)BCN-20A. Scale bars: 10 μm. Note that no apparent damage of the crystals is observed during the transformation from (Sc)BCN-20C to (Sc)BCN-20A.

Comparison of Unit Cells in the transformation from (Sc)BCN-20C to (Sc)BCN-20A

For comparison of the unit cell in the (Sc)BCN-20C system, (Sc)BCN-20C and (Sc)BCN-20A were intentionally indexed into the triclinic crystal systems. It should be noted that the unit cell of (Sc)BCN-20C and (Sc)BCN-20A can be transformed into monoclinic and trigonal lattices with higher symmetry, C2/c (No. 15) and $R\bar{3}c$ (No.167). For the valid crystallographic information, the single crystal X-ray crystallographic data for these three MOFs can be seen in **Tables S5-S7**.

Table S8. Comparison of the unit cell parameters of (Sc)BCN-20C, BCN-20C' and (Sc)BCN-20A.

	a (Å)	b (Å)	c (Å)	α (°)	β (°)	γ (°)	V (Å^3)
(Sc)BCN-20C	37.136	34.723	25.362	83.628	94.365	90	32406.1
(Sc)BCN-20C'	37.528	35.374	24.761	90	94.24	90	32780.7
(Sc)BCN-20A	35.914	28.682	22.987	91.344	106.436	90	22704.2

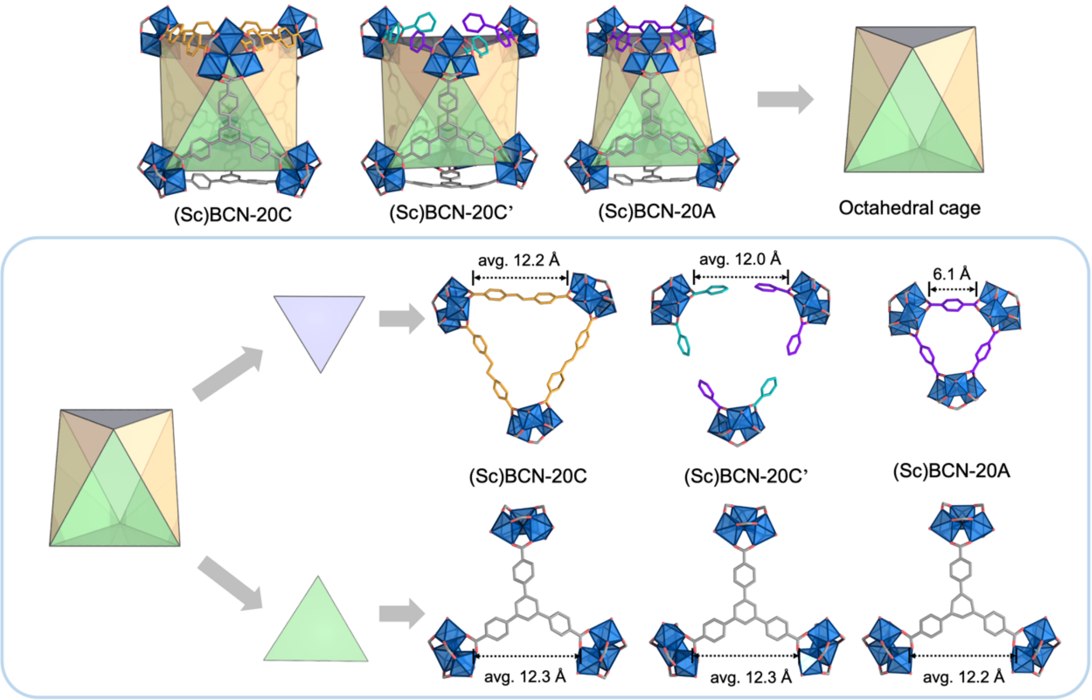

Figure S31. Schematic representation showing the single-crystal-to-single-crystal evolution from (Sc)BCN-20C to (Sc)BCN-20C’ to (Sc)BCN-20A, highlighting the changes in the octahedral cage.

References

1. Juanhuix, J.; Gil-Ortiz, F.; Cuní, G.; Colldelram, C.; Nicolás, J.; Lidón, J.; Boter, E.; Ruget, C.; Ferrer, S.; Benach, J. Developments in optics and performance at BL13-XALOC, the macromolecular crystallography beamline at the ALBA synchrotron. *J. Synchrotron Radiat.* **2014**, *21*, 679-689.
2. Kabsch, W. Integration, scaling, space-group assignment and post-refinement. *Acta Cryst. D* **2010**, *66*, 125-132.
3. Sheldrick, G. M. SHELXT-Integrated space-group and crystal-structure determination. *Acta Cryst. A* **2015**, *71*, 3-8.
4. Farrugia, L. J. WinGX and ORTEP for Windows: an update. *J. Appl. Cryst.* **2012**, *45*, 849-854.
5. Sheldrick, G. M. Crystal structure refinement with SHELXL. *J. Acta Cryst. C* **2015**, *71*, 3-8.
6. Dolomanov, O. V.; Bourhis, L. J.; Gildea, R. J.; Howard, J. A. K.; Puschmann, H. J. OLEX2: a complete structure solution, refinement and analysis program. *Appl. Cryst.* **2009**, *42*, 339-341.
7. Spek, A. L. Single-crystal structure validation with the program PLATON. *J. Appl. Crystallogr.* **2003**, *36*, 7-13.
8. A P Hammersley, *ESRF Internal Report*, **ESRF97HA02T**, ``FIT2D: An Introduction and Overview", (1997).
9. Park, J.; Feng, D.; Zhou, H.-C. Dual Exchange in PCN-333: A Facile Strategy to Chemically Robust Mesoporous Chromium Metal-Organic Framework with Functional Groups. *J. Am. Chem. Soc.* **2015**, *137*, 11801-11809.
10. Zou, L.; Feng, D.; Liu, T.-F.; Chen, Y.-P.; Yuan, S.; Wang, K.; Wang, X.; Fordham, S.; Zhou, H.-C. A versatile synthetic route for the preparation of titanium metal-organic frameworks. *Chem. Sci.* **2016**, *7*, 1063-1069.
11. Albalad, J.; Xu, H.; Gándara, F.; Haouas, M.; Martineau-Corcos, C.; Mas-Ballesté, R.; Barnett, S. A.; Juanhuix, J.; Imaz, I.; Maspoch, D. Single-Crystal-to-Single-Crystal Postsynthetic Modification of a Metal-Organic Framework via Ozonolysis. *J. Am. Chem. Soc.* **2018**, *140*, 2028-2031.
12. Guillerm, V.; Xu, H.; Albalad, J.; Imaz, I.; Maspoch, D. Postsynthetic Selective Ligand Cleavage by Solid-Gas Phase Ozonolysis Fuses Micropores into Mesopores in Metal-Organic Frameworks. *J. Am. Chem. Soc.* **2018**, *140*, 15022-15030.
13. Yang, Y.; Broto-Ribas, A.; Ortín-Rubio, B.; Imaz, I.; Gándara, F.; Carné-Sánchez, A.; Guillerm, V.; Jurado, S.; Busqué, F.; Juanhuix, J.; Maspoch, D. Clip-off Chemistry: Synthesis by Programmed Disassembly of Reticular Materials. *Angew. Chem. Int. Ed.* **2022**, *61*, e202111228.
